# More vs Less Frequent Follow-Up Testing and 10-Year Mortality in Patients With Stage II or III Colorectal Cancer: Secondary Analysis of the COLOFOL Randomized Clinical Trial

**DOI:** 10.1001/jamanetworkopen.2024.46243

**Published:** 2024-11-21

**Authors:** Henrik Toft Sørensen, Erzsébet Horváth-Puhó, Sune Høirup Petersen, Peer Wille-Jørgensen, Ingvar Syk

**Affiliations:** 1 Department of Clinical Epidemiology, Aarhus University Hospital and Aarhus University, Aarhus, Denmark; 2 Abdominal Disease Center, Bispebjerg Hospital and Danish Colorectal Cancer Group, København, Denmark; 3 Department of Surgery, Skåne University Hospital, Malmö, Sweden

## Abstract

**Importance:**

Although intensive follow-up of patients after curative surgery for colorectal cancer is common in clinical practice, evidence for a long-term survival benefit of more frequent testing is limited.

**Objective:**

To examine overall and colorectal cancer–specific mortality rates in patients with stage II or III colorectal cancer who underwent curative surgery and underwent high-frequency or low-frequency follow-up testing.

**Design, Setting, and Participants:**

This randomized clinical trial with posttrial prespecified follow-up was performed in 23 centers in Sweden and Denmark. The original study enrolled 2509 patients with stage II or III colorectal cancer from Sweden, Denmark, and Uruguay (1 center) who received treatment from January 1, 2006, through December 31, 2010, and were followed up for up to 5 years. The participants from Sweden and Denmark were then followed up for 10 years through population-based health registries. The 53 patients from Uruguay were not included in the posttrial follow-up. Statistical analysis was performed from March to June 2024.

**Interventions:**

Patients were randomly allocated to follow-up testing with computed tomography (CT) scans and serum carcinoembryonic antigen (CEA) screening at 6, 12, 18, 24, and 36 months after surgery (high-frequency group; 1227 patients), or at 12 and 36 months after surgery (low-frequency group, 1229 patients).

**Main Outcomes and Measures:**

The outcomes were 10-year overall mortality and colorectal cancer–specific mortality rates. Both intention-to-treat and per-protocol analyses were performed.

**Results:**

Of the 2555 patients who were randomly allocated, 2509 were included in the intention-to-treat analysis, of whom 2456 (97.9%) were included in this posttrial analysis (median age, 65 years [IQR, 59-70 years]; 1355 male patients [55.2%]). The 10-year overall mortality rate for the high-frequency group was 27.1% (333 of 1227; 95% CI, 24.7%-29.7%) compared with 28.4% (349 of 1229; 95% CI, 26.0%-31.0%) in the low-frequency group (risk difference, 1.3% [95% CI, −2.3% to 4.8%]). The 10-year colorectal cancer–specific mortality rate in the high-frequency group was 15.6% (191 of 1227; 95% CI, 13.6%-17.7%) compared with 16.0% (196 of 1229; 95% CI, 14.0%-18.1%) in the low-frequency group (risk difference, 0.4% [95% CI, −2.5% to 3.3%]). The same pattern resulted from the per-protocol analysis.

**Conclusions and Relevance:**

Among patients with stage II or III colorectal cancer, more frequent follow-up testing with CT scans and CEA testing did not result in a significant reduction in 10-year overall mortality or colorectal cancer–specific mortality. The results of this trial should be considered as the evidence base for updating clinical guidelines.

**Trial Registration:**

ClinicalTrials.gov Identifier: NCT00225641

## Key Points

**Question:**

Is intensive follow-up of patients after curative surgery for colorectal cancer effective in reducing 10-year mortality?

**Findings:**

In this posttrial prespecified secondary analysis of a randomized clinical trial that included 2456 patients with stage II or III colorectal cancer, follow-up testing with computed tomography scans and serum carcinoembryonic antigen screening was performed on 5 vs 2 occasions. More frequent testing did not result in a significant difference in between the high-frequency group and the low-frequency group in 10-year overall mortality (27.1% vs 28.4%) or cancer-specific mortality (15.6% vs 16.0%).

**Meaning:**

In patients with stage II or III colorectal cancer, increased frequency of follow-up testing did not reduce the 10-year mortality rate.

## Teaser

This prespecified secondary analysis of the COLOFOL randomized clinical trial examines overall and colorectal cancer–specific mortality rates in patients with stage II or III colorectal cancer who underwent curative surgery and underwent high-frequency or low-frequency follow-up testing.

## Introduction

Colorectal cancer is the third most common cancer worldwide, accounting for approximately 10% of all cancer cases, and is the second leading cause of cancer-related deaths globally. In 2022, more than 1.9 million cases were diagnosed worldwide and more than 900 000 colorectal cancer–related deaths occurred.^1,2^ Mortality is predominantly associated with metastatic disease, occurring synchronously and metachronously at equal rates.^3^

Two-thirds of patients with colorectal cancer present at tumor stage II or III.^4^ Most of these patients undergo curative resection and are eligible for subsequent surveillance screening. In most countries, patients undergo follow-up examinations to detect cancer recurrence, although programs differ considerably. The reasons for patient follow-up after curative colorectal cancer surgery include (1) to detect recurrence and adverse effects when curative treatment is still possible, thus improving survival; (2) to assess the quality of the primary treatment; (3) to detect metachronous tumors; and (4) to satisfy a patient’s desire for information about prognosis.^5^

Over the past several decades, approaches for treating disease recurrence, including resection of peritoneal, liver, and lung metastases as well as improved adjuvant and palliative chemotherapy, have improved patient outcomes and are continuously evolving.^6^ The improved treatments available for metastatic disease have led to an increased motivation to detect recurrences and thus the need to evaluate the potential benefit of providing more intensive vs less intensive surveillance programs after completion of the primary surgery.^7^ Systematic reviews and randomized clinical trials have provided somewhat inconclusive evidence regarding the survival benefit of more intensive vs less intensive surveillance programs.^8,9,10,11,12,13,14^

Based on this background, we conducted a randomized clinical trial (the COLOFOL trial) that included 2509 patients with stage II or III colorectal cancer.^7^ The patients were randomly allocated to either follow-up testing with computed tomography (CT) scan of the thorax and abdomen and serum carcinoembryonic antigen (CEA) screening at 6, 12, 24, and 36 months (1253 patients), or at 12 or 36 months after surgery (1256 patients). The primary outcomes of the trial were 5-year overall and colorectal cancer–specific mortality rates (trial protocol and statistical analysis plan in Supplement 1; eFigure in Supplement 2).

Although the trial did not provide entirely conclusive results, the findings revealed a small, potentially clinically important difference between the 2 groups. The 5-year mortality rates of these 2 groups were 13.0% for the high-frequency group and 14.1% for the low-frequency group (risk difference, 1.1% [95% CI, −1.6% to 3.8%]). The colorectal cancer–specific mortality rates were 10.6% for the high-frequency group compared with 11.4% among the patients in the low-frequency group in the intention-to-treat analysis (risk difference, 0.8% [95% CI, −1.7% to 3.3%]).

The longer-term findings from the COLOFOL study correspond with data from the Follow-up After Colorectal Surgery (FACS) trial of 1202 patients and a 2019 meta-analysis.^13,15^ The meta-analysis included data from 19 very heterogeneous studies,^15^ most of which were conducted in the era before the availability of advanced imaging techniques and/or multimodal treatment of metastatic disease. These studies featured a very high local recurrence rate and high frequency of metachronous primary tumors. A part of the meta-analysis that was based on 16 studies reported a nonsignificant difference in overall survival (hazard ratio [HR], 0.91 [95% CI, 0.80-1.04]). There were 1453 deaths among 12 528 participants in 15 studies. Although in absolute terms, the effect of intensive follow-up on overall survival was a mean of 24 fewer deaths per 1000 patients, the true effect could lie between 60 fewer to 9 more deaths per 1000 patients.

Based on the existing literature and the results of the initial analysis of the COLOFOL trial,^13,15^ there remains some uncertainty as to whether high-frequency follow-up is associated with a positive effect that can be translated into improved long-term outcomes based on substantially better strategies available to treat disease recurrence during the past several decades. Furthermore, positive effects might be more easily detected more than 5 years after the index procedure, given the improved rates of survival in metastatic disease.

Thus, given the advances in the treatment of metastatic disease, the effect of high-frequency follow-up might be detected beyond the 5 years included in the COLOFOL study. The earlier evaluation of this study also had fewer outcomes than expected from the a priori calculations of sample size that were performed when the study was designed. Based on information obtained from the high-quality health registries of the Nordic countries,^16^ we conducted an extended follow-up analysis (beyond 5 years) of the patients from Denmark and Sweden who were originally included in the COLOFOL study. We hypothesized that high-frequency follow-up screening with CT scan and CEA screening with a focus on the participants who had potentially been treated for recurrence would decrease the 10-year overall and colorectal cancer–specific mortality after curative surgery.

## Methods

The COLOFOL study group was established in 2004. Twenty-four recruitment centers in Denmark, Sweden, and Uruguay contributed patients to this study; patients received treatment from January 1, 2006, through December 31, 2010, and were followed up for up to 5 years. The study was approved by the National Ethical Committee in Denmark and the Ethics Committee, Uppsala, Sweden. Patients provided written informed consent. This study followed the Consolidated Standards of Reporting Trials (CONSORT) reporting guideline.

Inclusion criteria were as follows: patients who underwent surgical resection with curative intent for stage II or stage III colorectal adenocarcinoma (with or without adjuvant treatment), 75 years of age or younger; provision of written informed consent for participation, and a colon and rectum free of neoplasia verified by perioperative barium enema or a colonoscopy within 3 months after the initial surgery. Patients were allocated at random to follow-up testing with CT scan of the thorax and abdomen and serum CEA screening at 6, 12, 18, 24, and 36 months after surgery (ie, the high-frequency group; 1253 patients) or at 12 and 36 months after surgery (low-frequency group; 1256 patients) (eFigure in Supplement 2). Each center was required to follow up all study participants with surveillance examinations until 3 years after surgery according to the protocol and to report outcomes to the study’s coordinating center until 5 years after surgery. Clinically diagnosed recurrences and deaths were reported at the time of the event.

Exclusion criteria were as follows: a clinical diagnosis of hereditary nonpolyposis colorectal cancer or familial adenomatous polyposis, local resection of colorectal cancer (eg, a transanal endoscopic microsurgery procedure), a life expectancy of fewer than 2 years due to comorbid conditions (eg, cardiac disease, advanced multiple sclerosis with systemic complications, or liver cirrhosis), inability or refusal to provide informed consent, inability to comply with study requirements, inability to tolerate surgery for recurrence, other or previous malignant neoplasms (except for nonmelanoma skin cancer), or participation in another clinical trial that was incompatible with the COLOFOL study. For details on the study design, please refer to our previous publications.^5,7^

### Trial Follow-Up

All patients were tracked throughout the study by monitoring their inpatient and outpatient records and via linkage with national population and cancer registries (except in Uruguay, where only hospital data on 53 patients were available).^17,18^ Trial follow-up ended on December 31, 2015.

### Posttrial Follow-Up

The 10-year follow-up study ended on December 31, 2020. The outcomes of this prespecified extended follow-up analysis included 10-year overall and 10-year colorectal cancer–specific mortality rates. The outcomes were obtained from population-based cancer, death, pathology, and patient registries in Denmark and Sweden.^16^ Results presented in our earlier studies revealed that these outcome data are of high quality and can be obtained with complete follow-up.^19,20,21,22,23^ The posttrial analysis included 2456 of the original 2509 patients (97.9%); it was not possible to obtain follow-up information on the 53 patients (2.1%) from Uruguay.

### Statistical Analysis

Statistical analysis was performed from March to June 2024. Trial participants from Denmark and Sweden were followed up from the date of radical surgery for colorectal cancer until the date of the analyzed study outcome, date of dropout, date when lost to follow-up, or at 10-year follow-up, whichever came first. Information collected from the trial participants who were assigned to the high-frequency and low-frequency follow-up groups included sex, age, type of cancer, type of treatment, cancer stage, comorbidities, and lifestyle factors. Overall and colorectal cancer–specific mortality rates were analyzed both on an intention-to-treat and an as-treated per-protocol basis. The posttrial intention-to-treat analysis included 97.9% of participants (2456 of 2509) from the original COLOFOL trial. As a result, the posttrial analyses can be regarded as modified intention-to-treat analyses. Patients who withdrew informed consent or switched to another follow-up regimen remained linked to their randomized group for the intention-to-treat analysis but were excluded from the per-protocol analysis.

The Kaplan-Meier method and the log-rank test for between-group comparisons were used to analyze 10-year overall mortality among participants in the COLOFOL trial. The colorectal cancer–specific mortality rates were calculated using nonparametric methods for estimating cumulative incidence function with death from other causes treated as competing events. In addition, risk differences in the 10-year overall and colorectal cancer–specific mortality rates were calculated with corresponding 95% CIs using the Newcombe confidence limits method.

The main analyses were repeated for the subgroups of patients with stage II or III cancer and rectal and colon cancer. Two sensitivity analyses were performed to test the robustness of the 10-year risk estimates. First, the main analyses were repeated for trial participants from Denmark and Sweden, separately, and the overall and colorectal cancer–specific mortality rates were compared between the 2 countries. Second, Cox proportional hazards regression analysis was used to compute crude and adjusted HRs with 95% CIs for overall and colorectal cancer–specific mortality rates. The HRs were adjusted for age at the time of colorectal cancer surgery; sex; colorectal cancer type; cancer stage; comorbidities, including diabetes, cardiovascular disease, pulmonary disease, and cerebrovascular disease; preoperative radiotherapy; adjuvant chemotherapy; and smoking.

The level of significance was *P* < .05 with a 2-sided test. The analyses were conducted using SAS, version 9.4 (SAS Institute Inc).

## Results

Of the 2555 patients who were randomly allocated, 2509 were included in the intention-to-treat analysis, of whom 2456 (97.9%) were included in this posttrial analysis. The intention-to-treat analysis included 1227 patients (median age, 65 years [IQR, 59-70 years]; 693 male patients [56.5%] and 534 female patients [43.5%]) from the high-frequency follow-up group and 1229 patients (median age, 65 years [IQR, 59-70 years]; 662 male patients [53.9%] and 567 female patients [46.1%]) from the low-frequency follow-up group, corresponding to 55.3% of all eligible patients (2456 of 4445) (eFigure in Supplement 2). The median follow-up time for participants in the high-frequency group was 10 years (IQR, 9.1-10.0 years) and for those in the low-frequency group was 10 years (IQR, 8.9-10.0 years). The demographic and clinical characteristics of the trial participants are shown in Table 1. The participants in the high-frequency and low-frequency groups exhibited no major differences with respect to clinical characteristics and medical history.

**Table 1.** Demographic Characteristics and Clinical Prognostic Factors

Characteristic or factor	Patients, No. (%)			
	Intention-to-treat analysis		Per-protocol analysis	
	High-frequency group (n = 1227)	Low-frequency group (n = 1229)	High-frequency group (n = 1161)	Low-frequency group (n = 1160)
Sex				
Male	693 (56.5)	662 (53.9)	654 (56.3)	621 (53.5)
Female	534 (43.5)	567 (46.1)	507 (43.7)	539 (46.5)
Age group, y				
≤50	84 (6.8)	93 (7.6)	80 (6.9)	89 (7.7)
51-60	241 (19.6)	267 (21.7)	231 (19.9)	252 (21.7)
61-70	607 (49.5)	562 (45.7)	571 (49.2)	536 (46.2)
>70	295 (24.0)	307 (25.0)	279 (24.0)	283 (24.4)
Type of cancer				
Rectal	417 (34.0)	444 (36.1)	402 (34.6)	422 (36.4)
Right colon	346 (28.2)	349 (28.4)	319 (27.5)	324 (27.9)
Transverse colon	68 (5.5)	47 (3.8)	63 (5.4)	44 (3.8)
Left colon	409 (33.3)	412 (33.5)	388 (33.4)	391 (33.7)
Type of treatment				
Preoperative radiotherapy	242 (19.7)	269 (21.9)	233 (20.1)	252 (21.7)
Adjuvant chemotherapy	577 (47.0)	567 (46.1)	550 (47.4)	529 (45.6)
Cancer stage				
II (T3-4, N0, M0)	661 (53.9)	662 (53.9)	615 (53.0)	628 (54.1)
III (T1-4, N1-2, M0)	566 (46.1)	567 (46.1)	546 (47.0)	532 (45.9)
Medical history				
Diabetes	112 (9.1)	106 (8.6)	104 (9.0)	98 (8.4)
Cardiovascular disease	374 (30.5)	407 (33.1)	356 (30.7)	378 (32.6)
Pulmonary disease	77 (6.3)	66 (5.4)	69 (5.9)	60 (5.2)
Cerebrovascular disease	36 (2.9)	39 (3.2)	34 (2.9)	36 (3.1)
Other major disease	57 (4.6)	58 (4.7)	56 (4.8)	56 (4.8)
Lifestyle factors				
Current smoker	194 (15.8)	202 (16.4)	182 (15.7)	193 (16.6)
Consumes >3 alcohol drinks/d	58 (4.7)	52 (4.2)	53 (4.6)	50 (4.3)

**Figure 1.**
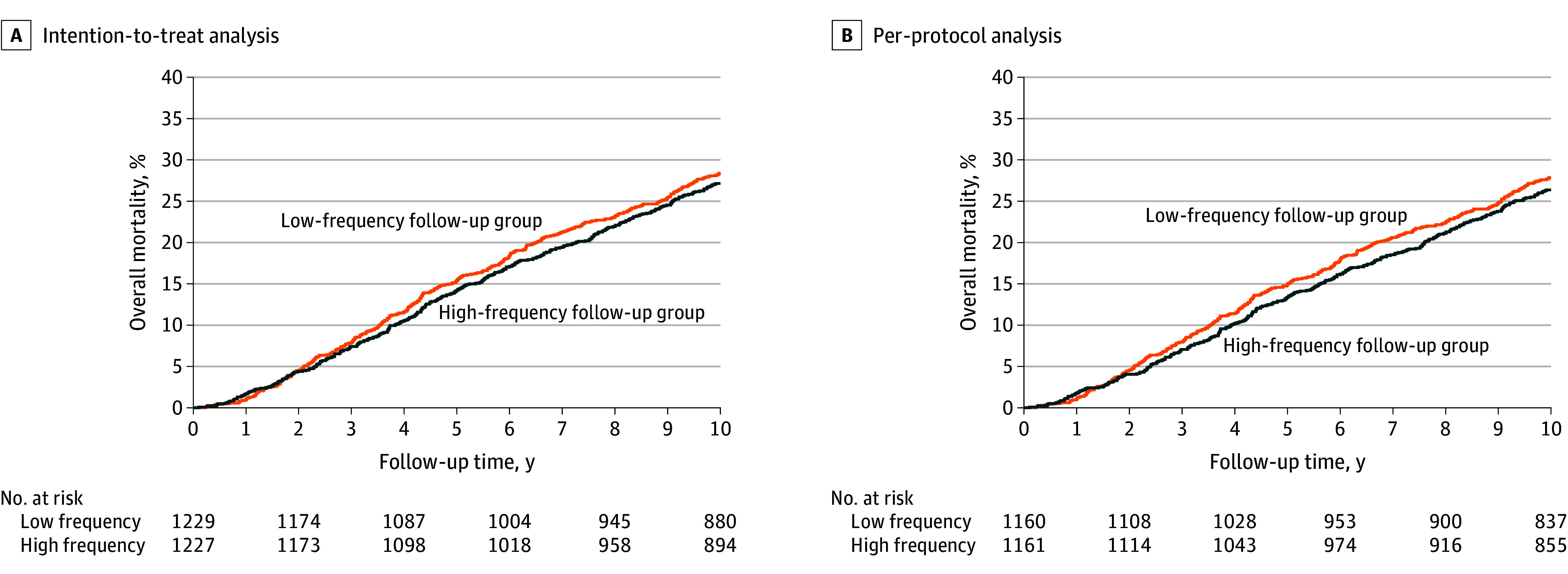
Overall Mortality Risk Based on the Time From Colorectal Cancer Surgery

Cumulative mortality curves are shown in Figure 1. In the intention-to-treat analysis, the 10-year overall mortality risk was 27.1% (333 of 1227; 95% CI, 24.7%-29.7%) among the patients in the high-frequency group vs 28.4% (349 of 1229; 95% CI, 26.0%-31.0%) among the patients in the low-frequency group (risk difference, 1.3% [95% CI, −2.3% to 4.8%]) (log-rank test of the between-group comparison, *P* = .46). In the per-protocol analysis, the 10-year mortality risk was 26.4% (306 of 1161; 95% CI, 23.9%-29.0%) among the patients in the high-frequency group vs 27.8% (323 of 1160; 95% CI, 25.4%-30.5%) among the patients in the low-frequency group (risk difference, 1.5% [95% CI, −2.1% to 5.1%]) (log-rank test of the between-group comparison, *P* = .38).

**Figure 2.**
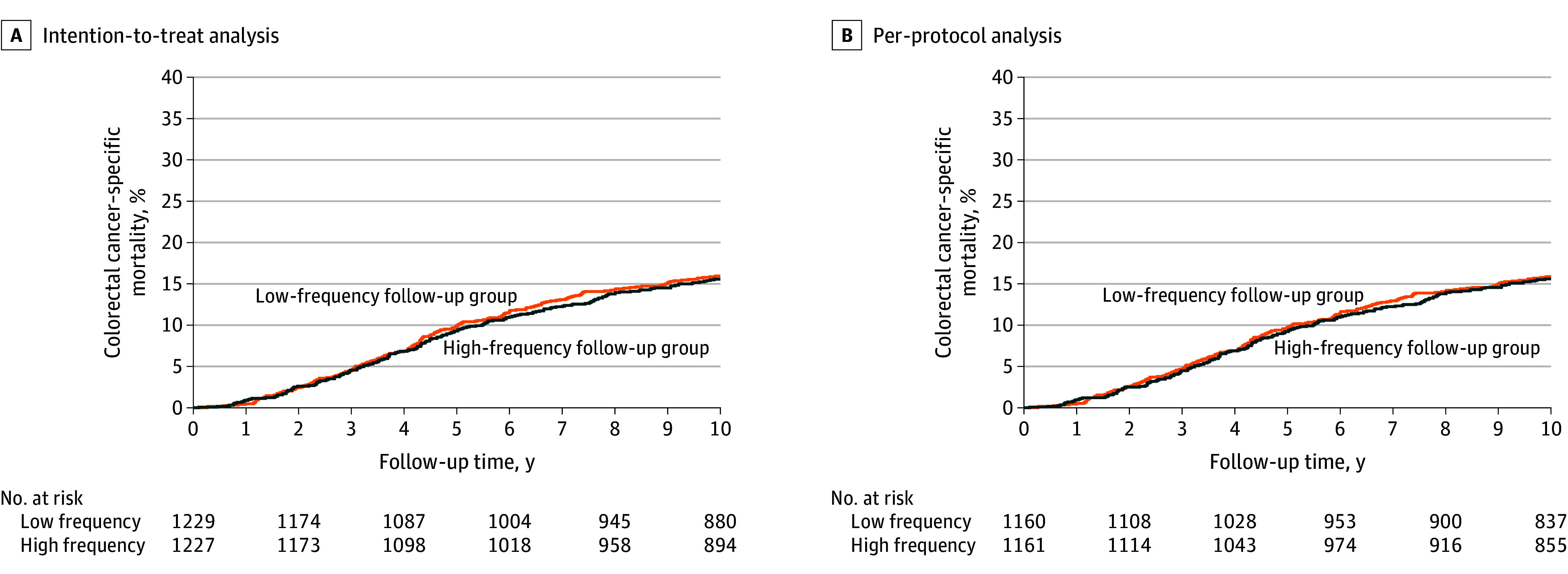
Cumulative Colorectal Cancer–Specific Mortality Risk Based on the Time From Colorectal Cancer Surgery

We observed no between-group differences in the 10-year colorectal cancer–specific mortality risk when comparing the intention-to-treat analysis with the per-protocol analysis (Figure 2). In the intention-to-treat analysis, the 10-year colorectal cancer–specific mortality risk was 15.6% (191 of 1227; 95% CI, 13.6%-17.7%) among the patients in the high-frequency group vs 16.0% (196 of 1229; 95% CI, 14.0%-18.1%) among the patients in the low-frequency group (risk difference, 0.4% [95% CI, −2.5% to 3.3%]) (log-rank test of the between-group comparison, *P* = .72). The same pattern was observed in the per-protocol analyses with a 10-year colorectal cancer–specific mortality risk of 15.6% (181 of 1161; 95% CI, 13.6%-17.7%) observed among the patients in the high-frequency group vs 15.9% (184 of 1160; 95% CI, 13.8%-18.0%) among the patients in the low-frequency group (risk difference, 0.3% [95% CI, −2.7% to 3.2%]) (log-rank test of the between-group comparison, *P* = .76). We observed no differences between the analyzed groups over the entire follow-up time.

The analyses repeated for the subgroups of patients with stage II or III cancer and those with rectal and colon cancer yielded no significant between-group differences (Table 2). In the intention-to-treat analysis, 10-year overall mortality was 22.4% (148 of 661; 95% CI, 19.4%-25.8%) among the patients with stage II cancer in the high-frequency group and 23.9% (158 of 662; 95% CI, 20.8%-27.3%) among the patients with stage II cancer in the low-frequency group (risk difference, 1.5% [95% CI, −3.1% to 6.0%]) and 32.7% (185 of 566; 95% CI, 29.0%-36.7%) among the patients with stage III cancer in the high-frequency group and 33.7% (191 of 567; 95% CI, 30.0%-37.7%) among the patients with stage III cancer in the low-frequency group (risk difference, 1.0% [95% CI, −4.5% to 6.5%]). The per-protocol estimates were similar to the intention-to-treat results.

**Table 2.** 10-Year Overall Mortality and 10-Year Colorectal Cancer–Specific Mortality Stratified by Cancer Stage and Cancer Type

Type of mortality	Risk estimate (95% CI), %		Risk difference (95% CI), %
	High-frequency group	Low-frequency group	
**10-Year overall mortality**			
Intention-to-treat analysis			
Stage II	22.4 (19.4 to 25.8)	23.9 (20.8 to 27.3)	1.5 (−3.1 to 6.0)
Stage III	32.7 (29.0 to 36.7)	33.7 (30.0 to 37.7)	1.0 (−4.5 to 6.5)
Rectal cancer	30.7 (26.5 to 35.4)	29.3 (25.3 to 33.8)	−1.4 (−7.5 to 4.7)
Colon cancer	25.3 (22.5 to 28.5)	27.9 (24.9 to 31.2)	2.6 (−1.8 to 6.9)
Per-protocol analysis			
Stage II	20.5 (17.5 to 23.9)	23.3 (20.1 to 26.8)	2.8 (−1.8 to 7.4)
Stage III	33.0 (29.2 to 37.1)	33.3 (29.4 to 37.5)	0.3 (−5.3 to 5.9)
Rectal cancer	30.4 (26.1 to 35.1)	27.7 (23.7 to 32.3)	−2.6 (−8.8 to 3.6)
Colon cancer	24.2 (21.4 to 27.5)	27.9 (24.8 to 31.3)	3.7 (−0.8 to 8.1)
**10-Year colorectal cancer–specific mortality**			
Intention-to-treat analysis			
Stage II	9.4 (7.3 to 11.8)	11.2 (8.9 to 13.7)	1.8 (−1.5 to 5.1)
Stage III	22.8 (19.4 to 26.3)	21.5 (18.2 to 25.0)	−1.3 (−6.1 to 3.6)
Rectal cancer	18.7 (15.1 to 22.6)	18.0 (14.6 to 21.7)	−0.7 (−5.9 to 4.5)
Colon cancer	14.0 (11.7 to 16.4)	14.8 (12.4 to 17.4)	0.8 (−2.6 to 4.3)
Per-protocol analysis			
Stage II	8.6 (6.6 to 11.0)	11.0 (8.7 to 13.6)	2.4 (−0.9 to 5.7)
Stage III	23.4 (20.0 to 27.1)	21.6 (18.2 to 25.2)	−1.8 (−6.8 to 3.2)
Rectal cancer	18.9 (15.2 to 22.9)	17.5 (14.1 to 21.3)	−1.4 (−6.6 to 3.9)
Colon cancer	13.8 (11.5 to 16.4)	14.9 (12.4 to 17.6)	1.1 (−2.5 to 4.6)

Sensitivity analyses showed no substantial differences between patients from the 2 countries (Denmark and Sweden; eTable 1 in Supplement 2). In the intention-to-treat analysis, no significant associations with the study outcomes were found in a Cox proportional hazards regression analysis controlling for covariates when 10-year overall or colorectal cancer–specific mortality rates were compared between patients in the high-intensity group vs patients in the low-intensity group (10-year overall mortality: adjusted HR, 0.93 [95% CI, 0.80-1.08]; and colorectal cancer–specific mortality: adjusted HR, 0.96 [95%CI, 0.78-1.17], respectively) (eTable 2 in Supplement 2).

## Discussion

In this posttrial follow-up of 2456 patients with colorectal cancer who underwent surgery with curative intent, we observed no significant differences in 10-year overall or 10-year colorectal cancer–specific mortality rates when the frequency of postoperative colorectal cancer follow-up was increased from 2 to 5 examinations during the first 3 years after surgery.

Our findings extend data from the initial trial, which did not reveal a significant reduction in 5-year overall or colorectal cancer–specific mortality. The original findings from this study were consistent with those from the FACS trial^13^ and a 2019 meta-analysis.^15^

The FACS trial included 1202 patients who had undergone curative surgery for colorectal cancer Dukes stages A to C in 39 hospitals across the United Kingdom.^13^ In the FACS trial, the patients were divided into 4 groups: minimum follow-up only, CEA testing only, CT scan only, or CEA testing and CT scan. Serum CEA levels were measured every 3 months for 2 years and then every 6 months for another 3 years. Computed tomography scans of the chest, abdomen, and pelvis were performed every 6 months for 2 years and then annually for 3 years. Those in the minimum follow-up and CEA testing only (ie, minimum follow-up) groups received only 1 CT scan during the 12 to 18 months of follow-up. The 3-year mortality was 18.2% in the combined intensive follow-up groups (ie, CT scan only or CEA testing and CT scan), compared with 15.9% in the combined minimum follow-up groups. The authors concluded that the survival advantage provided by any of these interventions was small, and that the trial lacked sufficient power to assess whether improved detection of treatable recurrences, such as that potentially achieved by intensive follow-up, might lead to reduced overall mortality.^14^

The 2019 meta-analysis did not exclude the possibility of a small overall survival benefit from intensified patient follow-up.^15^ The meta-analysis included 13 216 participants from 19 studies.

Both our trial and the FACS trial had lower mortality rates than expected. This outcome might be explained by the lower proportion of patients who underwent emergency procedures included in our study, with a better outcome than expected for patients who were recruited to a randomized clinical trial compared with those receiving routine clinical care.^24^ Furthermore, survival improved during the study period in the general population of patients with colorectal cancer in both Sweden and Denmark, probably due to improved adjuvant treatment and the availability of adjuvant treatment and surgical improvement in a few departments.^21,22,23^

Although neither trial could prove any survival benefit by more frequent follow-up in the randomized study groups, it might be that some high-risk subgroups could benefit. However, none of the trials were designed to evaluate this outcome, although the subgroup analysis of stage III tumors and an ad hoc analysis of the risk group with elevated CEA levels preoperatively and/or postoperatively showed no support to this assumption.^25^ Specially designed studies are needed to investigate this question.

### Limitations

Our study has several limitations. As in the initial trial, the turnover of responsible investigators and staff in many recruitment centers is a potential weakness because of the possibility of nonadherence to the protocol. This would most likely bias the trial findings toward null. However, we found that adherence to the follow-up protocol during the trial period was good in our study and similar to that described for the FACS trial.^13^ Similarly, it was not possible to blind patients and physicians to the frequency of follow-up examinations. Moreover, although it is well known that an intention-to-treat analysis tends to underestimate an effect, this bias was likely small as the per-protocol analysis yielded findings that were similar to those of the intention-to-treat analysis. Data on interventions performed for recurrences beyond the initial follow-up period may be relevant in long-term outcome analyses. A previous publication with information on all recurrences within 5 years, further treatment, and survival showed that 48% of all recurrences were treated with curative intent, without any significant difference between the study groups.^26^ Considering the high intervention ratio in both groups and that first recurrences beyond 5 years are rare, we find it less likely that low treatment aggressiveness of any potentially curative recurrence would explain the absence of any survival difference between the 2 study groups. Finally, although we were not able to follow up with 53 of the patients (2.1%) who came from Uruguay during the 5 to 10 years of follow-up, this small dropout rate is unlikely to explain our findings.

During the initial study period, Danish guidelines recommended follow-up protocols that corresponded with those offered by the follow-up frequency group of the curative trials. This is in contrast with Sweden, where no surveillance programs were used and the regimen varied from none to 5 years of annual follow-up. In 2014, the national guidelines in Sweden had no specific recommendations for follow-up for patients referred to the ongoing COLOFOL trial. Guidelines were changed in 2016 and advocated the use of the current trial’s low-frequency group regimen in the interim. The low-frequency follow-up regimen used in our trial is less intense than recommended in the guidelines provided by the National Comprehensive Cancer Network and the American Society of Clinical Oncology.^27,28,29^

## Conclusions

This secondary analysis of the COLOFOL randomized clinical trial found that, among patients with stage II or III colorectal cancer, more frequent follow-up testing with CT scan and CEA screening, compared with less frequent follow-up, did not result in a significant rate reduction in 10-year overall mortality or colorectal cancer–specific mortality. The results of this trial should be considered as the evidence base for updating clinical guidelines.

## Notes

Supplement 1. Trial Protocol and Statistical Analysis Plan

Supplement 2. **eTable 1.** 10-Year Overall Mortality and 10-Year Cancer-Specific Mortality in the Two Study Groups in Denmark and in Sweden **eTable 2.** Cox Proportional Hazards Regression for 10-Year Overall Mortality and 10-Year Colorectal Cancer–Specific Mortality in High-Frequency vs the Low-Frequency Groups **eFigure.** Patient Flow Through the COLOFOL Trial

Supplement 3.
Nonauthor Collaborators

Supplement 4.
Data Sharing Statement
