# Supplementary material for: More vs Less Frequent Follow-Up Testing and 10-Year Mortality in Patients With Stage II or III Colorectal Cancer: Secondary Analysis of the COLOFOL Randomized Clinical Trial
Source: JAMA Netw Open. 2024 Nov 21;7(11):e2446243. doi: 10.1001/jamanetworkopen.2024.46243 (PMC11582930; doi:10.1001/jamanetworkopen.2024.46243)
Supplement: Supplement 1. — Trial Protocol and Statistical Analysis Plan [file jamanetwopen-e2446243-s001.pdf]

# Online Supplemental Material to JAMA17-9394
# Effect of More vs Less Frequent Follow-Up Testing on Total and Cancer-Specific Mortality in Patients with Stage II-III Colorectal Cancer: The COLOFOL Randomized Clinical Trial

**Contents**

**1. COLOFOL Study eProtocol** ........ 2
**2. eStatistical Analysis Plan** ........ 54

# 1. COLOFOL Study eProtocol

**A pragmatic study to assess the frequency of surveillance tests after curative resection in patients with stage II and III colorectal cancer**
**– a randomised multicentre trial**
–

Running title:
Assessment of frequency of surveillance after curative resection in patients with stage II and III colorectal cancer.

*Acronym:* **COLOFOL**

*by*

*Peer Wille-Jørgensen, MD, Dr Med Sci.*
*Søren Laurberg, MD, Dr Med Sci*
*Henrik Toft Sørensen, MD, Dr Med Sci*
*Lars Påhlman, MD, Dr Med Sci*

on behalf of the COLOFOL study group

Correspondence to:

Peer Wille-Jørgensen, M.D., Dr. Med. Sci.
Department of Surgical Gastroenterology K
H:S - Bispebjerg Hospital
DK-2400 Copenhagen NV
DENMARK
E- Mail:  pwj01@bbh.hosp.dk

AIM

To conduct a prospective multicentre randomised study comparing total mortality, cancer-specific mortality, recurrence-free survival, economic cost effectiveness, and quality of life (QOL) in patients having two different schedules for follow-up after radical resection for colorectal cancer.

BACKGROUND

The value of follow-up programmes after radical surgery for colorectal cancer has been a controversial issue for many years, and the scientific evidence supporting it remains sparse. Many cohort and case-control studies have supported the effectiveness of follow-up (Rosen *et al*., 1998) (Figure 1 – lower case)**,** but, until recently, randomised controlled trials have reported only ambiguous findings regarding the efficacy of follow-up on mortality (Kievit & Bruinvels, 1995). Nonetheless, despite the sparse evidence, follow-up programmes are being used in most clinics treating colorectal cancer patients (Mella *et al*., 1997).

The reasons for follow-up include:

- to obtain a better overall survival.
- for scientific purposes
- for psychological reasons

There is no doubt that the outcome of follow-up programmes can be considered from both efficacy and cost perspectives (Kievit, 2002).

Recently, two systematic reviews with meta-analyses have been published investigating the same five randomised controlled trials (Renehan *et al*., 2002; Jeffery *et al*., 2002). The conclusions of these reviews were the same: more intensive follow-up leads to lower mortality than sporadic or less intensive follow-up. Subsequently, another randomised study was published confirming this conclusion (Secco *et al*., 2002). We have conducted a meta-analysis of all the randomised trials, as shown in Figure 1 (upper case). We found a highly significant effect of more intensive follow-up compared with less intensive follow-up (Peto Odds Ratio=0.62, 95% confidence interval:0.51-0.76, $P < 0.001$).

However, these meta-analyses do create problems, mainly due to the heterogeneity in both the intense and less intense follow-up regimens. The individual trials used different control modalities and, in fact, the intensity of control in one study could be considered more aggressive than the "intensive" group in other studies. The quality of the trials has also been questioned, as a high (and probably not generalisable) number of local recurrences was seen in the two studies which showed the highest effect of intensive follow-up (Secco *et al*., 2002; Pietra *et al*., 1998). The results of these meta-analyses should, thus, be viewed with caution.

Further, the meta-analyses are not able to identify either which follow-up modality to use, or the intensity (*i.e*., investigation intervals) of the follow-up. Although some authors claim that we know enough to make evidence-based recommendations (Smith & Bear, 1998) this claim is clearly arguable – a point of view that is supported by the debate that followed Renehan´s paper in *British Medical Journal* in April 2002 (see www.bmj.com/cgi/eletters/324/7341/813#23550)

Nonetheless, the published studies do imply that finding extraluminal recurrences (local recurrence after rectal cancer and liver metastases after colorectal cancer) mainly causes the obtainable benefit. The search for intraluminal recurrence does not alter the mortality. As the incidence of local recurrence after rectal cancer is rapidly declining due to better surgical techniques and the use of adjuvant treatment, the effect of looking for local recurrence after rectal cancer might be limited. A subgroup analysis of three studies looking for liver metastases shows an effect on

survival in one of the meta-analyses (Figure 2) (Jeffery *et al*., 2002). Moreover, the preoperative work-up for occult liver metastases was not routine when the referred trials where conducted.

In conclusion, the effectiveness of high-volume follow-up programmes after radical surgery for colorectal cancer on overall survival is still not sufficiently elucidated to create evidence-based guidelines. Despite this the Current Oncological Practice (COP) is moving towards high intensity follow-up programmes (Figueredo et al 2003, Worthington et al 2004, Anthony et al 2004). A Minimal acceptable practice (MAP) might be just as good. It, thus, seems justified to perform a randomised controlled trial randomising patients to high- or low-volume control programmes.

## Most used methods for control

*Luminal recurrence:*
Endoscopy is the chosen method, but luminal recurrence is seldom and programmes looking for luminal recurrence show no effect (Renehan *et al*., 2002)

*Local recurrence after rectal cancer:*
Endorectal ultrasound (US) has a high sensitivity and specificity (Löhnert *et al*., 2000), especially when combined with guided biopsy (Hünerbein *et al*., 2001). MR-imaging (MRI) is perhaps even more sensitive, but expensive (Pegios *et al*., 2002).

*Liver metastases:*
Average sensitivity for US is 55%, for CT 72%, MDCT 96% (Acta Rad. vol 46, no 1, feb. 2005, 9-15), for MRI 76%, and for PET 90%. Of these, PET and MRI are not generally available.

*General recurrence:*
PET is probably the most sensitive of detecting dissiminated disease Monitoring of carcinoembryonic antigen (CEA) remains controversial (Lennon *et al*., 1994), but might be justified, if related to perioperative measurements (Renehan *et al*., 2002; Li Destri *et al*., 1998).

## STUDY PROCEDURES

*Recruitment and Eligibility*

Centres will be recruited from all over Scandinavia, the UK, Poland, Hungary, Uruguay and Holland. At each participating centre, consecutive patients receiving radical surgery for colorectal cancer (emergency or elective) will be considered for inclusion. Those who are ineligible or who refuse consent will be followed in an observational follow-up design.

Inclusion criteria:

- Radical elective or emergency surgery (R0-resection) for colorectal adenocarcinoma – with or without adjuvant treatment, and
- Age $\leq$ 75 years, and
- Provision of written informed consent for participation, and
- “Clean colon” verified by perioperative barium enema or colonoscopy last 3 months post-surgery, and
- Tumour stage:II-III ($T_{any}$ $N_{1-2}$ $M_0$, $T_{3-4}N_{any}M_0$, Dukes´ B - C) as staged at the pretreatment staging procedure (clinical staging)

Pre- or postoperative chemotherapy and/or radiation therapy is allowed.

Exclusion criteria:

- A clinical diagnosis of HNPCC (non hereditary polyposis colorectal cancer) or FAP (familial polyposis coli),
- Local resection for colorectal cancer (*e.g.*, TEM-procedure),
- Life-expectancy less than 2 years due to concurrent disease (*e.g.*, cardiac disease, terminal multiple sclerosis, liver cirrhosis),
- Inability to provide informed consent or refusal to do so,
- Inability to comply with the control or intense follow-up programme,
- Participation in other clinical trials interfering with the control-programmes
- Other or previous malignancies (except for non-melanoma skin cancer)

Informed consent (written) is obtained within 30 days after the primary tumour classification has been obtained. Patients refusing to participate should be asked for consent to be followed in the cohort, using data on their actual control-programme and their clinical course.

**Randomisation and treatments**

Randomisation will take place by Internet from a central randomisation unit (appendix 12) placed at Department of Clinical Epidemilogy in Århus Denmark. Randomisation will be stratified according to tumour stage and clinical centre. Randomisation will be blocked in variable groups of which the size will be kept secret for the participating centres. The allocation procedure should be concealed to the deliverers of treatment.

**Study Follow-up Regimens**

CEA will (if possible) be measured on all colorectal cancer patients before surgery or preoperative adjuvant therapy, and one month after the completion of primary treatment. All patients should have “clean colon” within 3 months perioperatively, and at least one imaging procedure (Ultrasound, MRI, or CT) of the liver and X-ray of the lungs perioperatively. In both groups, an unlimited number of endoscopies are allowed. Interval diagnostic evaluation will be allowed for all subjects presenting symptoms.
A clinical visit 4-6 weeks after operation is recommended for information and allocation.

*1: Low frequency follow-up regimen*

- CEA preoperatively and one month postoperatively – a missing preoperative CEA is not considered an exclusion criteria, and will be handled as missing data.
- CEA, mulitislice CT/ or MRI of the liver and X-ray/CT of the lungs 12 and 36 month after surgery. Patients are instructed to contact their reference centre if symptoms of recurrence occur.
- If recurrent disease is suspected, a standardised work-up for diagnosis and treatment will be applied (appendix 2-3)

*2: High frequency follow-up regimen*

- CEA preoperatively and one month postoperatively – a missing preoperative CEA is not considered an exclusion criteria, and will be handled as missing data.
- CEA, multislice hepatic CT, or MRI and X-ray or CT of the lungs at 6, 12, 18, 24, and 36 months. Patients are instructed to contact their reference centre if they experience any symptoms of recurrence.

- If recurrent disease is suspected, a standardised work-up for diagnosis and treatment will be applied (appendix 2-3)

Patients with newly elevated CEA in whom recurrence cannot be found at other investigations could be sent for PET-scanning (if available). If this does not disclose metastatic disease, it should be repeated after 3 months (if the CEA is still elevated). For algorithm see appendix 2.

At each follow-up visit, a blood sample is taken for storage (-80°) for possible later analyses for prognostic or diagnostic factors.

In both groups, an unlimited number of endoscopies are allowed, but should be performed after the same schedule in each randomisation-group two groups. The same goes for CT/MRI/US of the pelvis for recurrence after rectal cancer. The local programmes in this respect should be registered centrally, and divergations from this programme should be registered in the CRF. Interval control should be performed in case of symptoms, and the procedures used should be according to the guidelines in appendix 2. These interval controls should be registered on the CRF.
It is allowed to call patients for extra clinical follow- up after 48 and 60 months, as long as the programme is even in the two randomisation arms.

*Quality of examinations*
Before entering a trial, the individual centre should prove the high quality of their control-examinations. The central steering group's diagnostic board should approve five consecutive examples. Guidelines for examinations are listed in Appendix 1.

**Patient treatment if recurrence**

If recurrence is suspected or verified, the patient-case should be evaluated in a local (country- or county separated) MDT conference, in order to decide and offer the best available treatment for the patient (salvage surgery, palliative chemotherapy and/or radiotherapy, no treatment). These groups also form the local steering-groups for the study. They should preferably include a surgeon, a radiologist, and an oncologist.

An algorithm for treatment of recurrence is found in Appendix 3.

**Efficacy parametres**

The primary effect-parameter of the study will be total mortality and cancer-specific mortality after 5 years; the secondary endpoint will be time to diagnosis of recurrence (*i.e.*, recurrence-free survival).

**Data collection**

In order to track all potentially eligible patients, a system for reporting all patients diagnosed with colorectal adenocarcinoma in the pathological departments of participating hospitals should be in place at each participating centre. All patients having radical surgery and who fulfil the inclusion criteria are to be reported to the secretariat. The reason for not including individual patients is to be reported. Case-record forms (CRF) (Appendix 6) for individual patients are to be filled in at the individual centres. Reports should be collected centrally after every planned or non-planned control. All registrations (CRF) are communicated centrally by means of an internet-based database with the central server placed at The Department of Clinical Epidemiology in Århus, Denmark after each visit (appendix 12).

Patients refusing to participate should be followed according to the individual departments' local guidelines. Each control-visit among patients in this group should be reported, if consent for observational follow-up is obtained.

If economically possible, each centre will receive a monitoring visit at least once a year.

The following data will be obtained on all randomised subjects and those who give consent for observational study:

- Baseline demographic, clinical, and lifestyle factors (date of birth, major chronic illnesses, smoking, alcohol intake, medications, etc);
- Results of all diagnostic evaluations for metastatic disease during the initial evaluation;
- Description of initial surgical, adjuvant, and radiation treatment;
- Postoperative final staging
- Findings from all cancer surveillance tests obtained after diagnosis;
- Detailed description of all cancer treatment applied after randomisation;
- Quality of life (QOL) assessment by telephone interview at 1, 3, and 5 years after randomisation. A standardised and evaluated QOL-instrument that takes follow-up regimens into consideration will be used. (See Appendix 3.)

**Statistical analysis and power**

Results will be evaluated both on an intention-to-treat basis and on an as-treated (per protocol) basis. Patients who withdraw their informed consent and, thus, change their control-programme remain in their allocation group when evaluated on the intention-to-treat basis, and are excluded when evaluated on the fulfilled protocol basis. Non-randomised patients and patients withdrawing their informed consent will be analysed separately.

On the basis of the results listed in Figure 1, an estimate of 5 years' mortality at 60% and a MIREDIF (Minimal Relevant Difference) of 6% seem justified. With a risk of type 1 error at 5% and type 2 error of 15%, about 1,100 patients should be randomised to each group. With an expected dropout rate of about 20%, the planned number of randomised patients should be 2,500.

Survival data will be analysed according to the Kaplan-Meier method and comparison between groups will be performed with the log-rank method. Binominal data will be analysed with Chi-square statistics and continuous data with Mann-Whitney U test.

No interim analyses are planned, as this would stop inclusion for several years.

Level of significance two-sided $p < 0.05$.

**Ethical considerations**

The meta-analyses provide no reason for a firm conclusion on the effectiveness of specific follow-up programmes, although arguably the studies “consistently” suggest that more follow-up (of some sort) is better than no follow-up at all. The question is how often patients have to be checked. Community equipoise exists due to the uncertainty of the regimens. There might be problems with patients' and doctors' equipoise, as patients report a strong preference for pre-scheduled follow-up (Stiggelbout *et al.*,1997), but the opposite feelings also exist among patients. In a similar investigation among patients with breast cancer, 66.5% of the women accepted randomisation into follow-up in the primary or hospital sector.

Patients in the intervention group might appear to be offered more intense treatment. It is doubtful whether this is of benefit for the individual patient, considering the psychological stress the intensive follow-up programme and the possible side effects of extra therapy will imply for the patient.

Patients refusing to participate will be followed according to the individual departments' local guidelines. Patients who refuse to participate are followed in the database. Consent for this is not needed, as this is a part of the general record keeping.

All participants must give informed consent for participation. This can be withdrawn at any time.

The demands in the Declaration of Helsinki are met.

**Organisation**

The study will be undertaken under the auspices of a steering committee elected among the persons in the study group. The steering group should consist of prefereable 2 persons from each participating country. The monitoring groups will be appointed among people not directly involved in the investigation.

Centres will be recruited from all over Denmark, Sweden, Poland and Uruguay. Due to the patients' preference for pre-scheduled follow-up, a low recruitment rate can be expected. Each centre should be able to recruit at least 50 patients within two years. This requires at least 50 participating centres.

Each centre will appoint a principal investigator who will be responsible for local administration and recruitment of diagnostic departments (Radiology, Nuclear Medicine, Ultrasound departments). Each country appoints a monitoring committee, and a committee for evaluation of patients suspected of recurrence. Each centre is recommended to appoint a study nurse for running the study.

A central secretariat will be placed in a suitable institution (pt at Cochrane Colorectal Cancer Group at Bispebjerg Hospital, Copenhagen, Denmark) and is staffed with a full-time secretary and preferably with a half-time academic employee. The secretariat will collect a log of all included patients, monitor the individual centres for inclusion of eligible patients on a daily basis, and serve as the central allocation unit. If a centre does not recruit at least 30% of eligible patients, the centre should be withdrawn from the study.

**Economics**

No industrial sponsors can be expected to volunteer in this project. Funds will be sought from private and official funds in the participating countries. Funds from the EU will also be considered. The central steering group will support applications for local support.

If economically feasible each included patient should be reimbursed with 125 EUROs to be given to the individual department. Total expense is estimated to be 375,000 EUROs.

Centres will cover their own expenses for the diagnostic tests.

Organisational expenses are also expected for the secretariat and data handling unit (estimated at 100,000 EUROs/year), for travel and meeting expenses, for local monitoring and secondary treatment groups (estimated at 150,000 EUROs/the whole study). Expenses for the diagnostic investigations and study-nurses in the individual centres are to be met directly by the centres. With

2 years of recruitment and 5 years of follow-up, the total requirement for funds will be about 1,250,000 EUROs.

The Nordic Cancer Union was sought for grants for starting and detailed planning of the study by May 15, 2003; 25.000 EUROs were allowed to cover the planning phase and a feasibility pilot study.

The Danish Cancer Society has granted 800.000 dkr. for use in 2007/2008. And the Danish Agency for Science, Technology and Innovation donated 1.082.550 dkr. to be used in 2008-2010.

**Side-protocols**

Such are allowed in the respect that they do not interfere with the main project. At this stage, the following side-protocols are planned:

- Cost effectiveness-analyses stratified for each participating country;
- Sampling of blood and tissue for risk-factor analysis;
- Quality of life for included patients
- Observational study of survival and risk factors after salvage surgery for recurrence.

Private companies migth be involved in some local side-protocols. This could create conflict of interests. Local projects on COLOFOL patients will be performed with no responsibility from the steering group, but will have to be approved by the steering group, ensuring no interference with the main project occurs. The side-protocols have to develop their own patient information and ethical considerations.

**Time schedule**

2003-4: Planning, fund-raising, final protocol, case record forms, ethical committees, recruitment of centres, feasibility studies.
2005-8: Inclusion of patients
2013: Last patient followed-up for 5 years.

**Publication**

The Steering Group will appoint a writing committee to report the results on behalf of the COLOFOL-group. All investigators will be eligible. All investigators will be listed by name at the end of the presenting paper.

**Amendments to the original protocol decided by the steering group after initiation of trial:**

Regarding **Data collection**:

The initial request of the reporting of all non-included eligible patients proved difficult to insure in all participating centers, so a comparison of study and source population will be explored in a separate paper.

The Quality of life measurement will be conducted as a side protocol and reported as a separate paper.

The Cost effectiveness will be reported as a separate paper.

Regarding **Efficacy parameters**:

As evidence suggested that the 3 year effect-parameter of total mortality and cancer-specific mortality to be an excellent surrogate to 5 years, the steering decided in September 2012 to publish both 3 and 5 years total mortality and cancer-specific mortality, along with the secondary endpoint of recurrence-free survival.

Regarding **Organisation**:

The initial requirement that If a centre does not recruit at least 30% of eligible patients, the centre should be withdrawn from the study and that each centre should be able to recruit at least 50 patients within two years,  were explicitly based on at least 50 participating centres. Despite continues effort neither the UK, Holland nor Hungary will be participating, so an adjustment is needed to achieve the planned number of randomised patients.  The Steering Group decided in September 2010 to impose cut-of off >20 included patients to insure quality of the management at the participating department in Denmark, Ireland, Poland, Sweden and Uruguay.

Regarding T**ime schedule**:

The schedule  will adjusted according to the prolonged inclusion period, so that inclusion are to be completed by the end of 2010 and the completion of the last patient follow-up for 5 years by the end 2015/early 2016.

**Appendices**

1. Recommended techniques for diagnostic investigations and certification
2. Algorithm for positive findings in the follow-up
3. Guidelines for handling of detected recurrences in the study groups
4. QOL-instrument
5. Participating Centres and persons
6. Registration forms
7. Steering group
8. Monitoring committee (to be elected in autumn 2004)
9. Patient information and lay person protocol (In local languages)
10. Databases and randomisation procedures
11. Approval from ethical committees
12. Flow-chart and inclusion chart

**REFERENCES**


Anthony T, Simmang C, Hyman N, Buie D, Kim D, Cataldo P et al. Practice parameters for the surveillance and follow-up of patients with colon and rectal cancer. Dis Colon Rectum 2004; 47(6):807-817.

Figueredo A, Rumble RB, Maroun J, Earle CC, Cummings B, McLeod R et al. Follow-up of patients with curatively resected colorectal cancer: a practice guideline. BMC Cancer 2003; 3(1):26.

Hunerbein M, Totkas S, Moesta KT, Ulmer C, Handke T, Schlag PM. The role of transrectal ultrasound-guided biopsy in the postoperative follow-up of patients with rectal cancer. *Surgery* 2001; 129(2): 164-9

Jeffery GM, Hickey BE, Hider P. Follow-up strategies for patients treated for non-metastatic colorectal cancer (Cochrane Review). In: The Cochrane Library, Issue 4, 2002. Oxford: Update Software.

Kievit J. Follow-up of patients with colorectal cancer: numbers needed to test and treat. *Eur J Cancer* 2002; 38: 986-99.

Kievit J, Bruinvels DJ. Detection of recurrence after surgery for colorectal cancer. *Eur J Cancer* 1995; 31A: 1222-5

Kinkel K, Lu Y, Both M, Warren RS, Thoeni RF. Detection of hepatic metastases from cancers of the gastrointestinal tract by using non-invasive imaging methods (US, CT, MR Imaging, PET): a meta-analysis. *Radiology* 2002; 224: 748-56.

Lennon T, Houghton J, Northover J. Post-operative CEA monitoring and second look in colorectal cancer. trial results *Br J Cancer* 1994; 70 (supp XXII): 16.

Li Destri G, Greco S, Rinzivillo C, Racalbuto A, Curreri R, Di Cataldo A. Monitoring carcinoembryonic antigen in colorectal cancer. Is it still useful?. *Surg Today* 1998; 28: 1233-6.

Löhnert NMSS, Doniec JM, Henne-Bruns D. Effectiveness of endoluminal sonography in the identification of occult local rectal cancer recurremces. *Dis Colon Rectum* 2000; 43: 483-91.

Mella J, Radcliffe AG, Datta SN, Steele RJC, Biffin A, Stamatakis JD. Surgeons' follow-up practice after resection of colorectal cancer. *Ann Roy Coll Surg* 1997; 79: 206-9.

Pegios W, Hunerbein M, Schroder R, Wust P, Schlag P, Felix R, Vogl TJ. Comparison between endorectal MRI (EMRTI) and endorectal sonography (ES) after surgery or therapy for rectal tumors to exclude recurrent or residual tumor. *Rofo Fortschr Geb Rontgenstr Neuen Bildgeb Verfahr* 2002; 174: 731-7

Pietra N, Sarli L, Costi R, Ouchemi C, Grattarola M, Peracchia A. Role of follow-up in management of local recurrences of colorectal cancer. *Dis Colon Rectum* 1998; 41: 1127-33.

Renehan AG, Egger M, Saunders MP, O'Dwyer ST. Impact on survival of intensive follow up after curative resection for colorectal cancer: systematic review and meta-analysis of randomised trials. *BMJ* 2002; 324(7341): 813-21

Rosen M, Chan L, Beart RW, Vukasin P, Anthone G. Follow-up of colorectal cancer. A meta-analysis. *Dis Colon Rectum* 1998; 41:1116-26.

Secco GB, Fardelli R, Gianquino D, Bonfante P, Baldi E, Ravera G, Derchi L, Ferraris R. Efficacy and cost of risk-adapted follow-up in patients after colorectal cancer surgery: a prospective randomised and controlled trial. *EJSO* 2002; 28: 418-23.

Smith TJ, Bear HD. Standard follow-up of colorectal cancer patients: finally we can make practice guidelines based on evidence. *Gastroenterology* 1998; 114: 211-3.

Stiggelbout AM, De Haas JCJM, Vree R, van de Velde CJH, Bruijninckx CMA, van Groningen K, Kievit J. Follow-up of colorectal cancer patients: quality of life and attitudes towards follow up. *Br J Cancer* 1997; 75: 914-20.

Worthington TR, Wilson T, Padbury R. Case for postoperative surveillance following colorectal cancer resection. ANZ J Surg 2004; 74(1-2):43-45.

**Figure 1**

**Comparison: 01 Death from colorectal cancer**
**Outcome: 01 Death after colorectal cancer with intensive or sporadic follow-up**

| Study | Intensive n/N | Less Intensive n/N | Peto OR (95%CI Fixed) | Weight % | Peto OR (95%CI Fixed) |
|---|---|---|---|---|---|
| 01 Randomised controlled trials | | | | | |
| Kjeldsen | 88 / 290 | 100 / 307 | | 18.3 | 0.90[0.64,1.27] |
| Makala | 23 / 52 | 27 / 54 | | 3.8 | 0.80[0.37,1.70] |
| Ohlson | 15 / 53 | 22 / 54 | | 3.5 | 0.58[0.26,1.28] |
| Pietra | 28 / 104 | 43 / 103 | | 6.7 | 0.52[0.29,0.92] |
| Schoemaker | 43 / 167 | 55 / 158 | | 9.8 | 0.65[0.41,1.05] |
| Secco A | 53 / 108 | 56 / 84 | | 6.6 | 0.49[0.28,0.87] |
| Secco B | 16 / 84 | 35 / 64 | | 4.7 | 0.21[0.11,0.41] |
| Subtotal(95%CI) | 266 / 858 | 338 / 824 | | 53.4 | 0.62[0.51,0.76] |
| Test for heterogeneity chi-square=15.82 df=6 p=0.015 | | | | | |
| Test for overall effect z=-4.59 p<0.00001 | | | | | |
| 02 Observational studies | | | | | |
| Bergamaschi | 168 / 322 | 282 / 478 | | 27.0 | 0.76[0.57,1.01] |
| Eckhardt | 103 / 368 | 53 / 139 | | 12.3 | 0.62[0.41,0.95] |
| Ovaska | 25 / 88 | 77 / 124 | | 7.4 | 0.26[0.15,0.45] |
| Subtotal(95%CI) | 296 / 778 | 412 / 741 | | 46.6 | 0.61[0.49,0.76] |
| Test for heterogeneity chi-square=11.55 df=2 p=0.0031 | | | | | |
| Test for overall effect z=-4.50 p<0.00001 | | | | | |
| Total(95%CI) | 562 / 1636 | 750 / 1565 | | 100.0 | 0.62[0.53,0.71] |
| Test for heterogeneity chi-square=27.39 df=9 p=0.0012 | | | | | |
| Test for overall effect z=-6.43 p<0.00001 | | | | | |

.1 .2 1 5 10

Favours treatment Favours control

**Figure 2**

**Comparison: Liver imaging versus no liver imaging**
**Outcome: Mortality**

| Study | Expt n/N | Ctrl n/N | Peto OR (95%CI Fixed) | Weight % | Peto OR (95%CI Fixed) |
|---|---|---|---|---|---|
| Makela 1995 | 23 / 52 | 27 / 54 | | 22.3 | 0.80 [0.37,1.70] |
| Ohlsson 1995 | 15 / 53 | 22 / 54 | | 20.4 | 0.58 [0.26,1.28] |
| Schoemaker 1998 | 43 / 167 | 55 / 158 | | 57.3 | 0.65 [0.41,1.05] |
| Total (95%CI) | 81 / 272 | 104 / 266 | | 100.0 | 0.66 [0.46,0.95] |
| Chi-square 0.33 (df=2) Z=2.23 | | | | | |

**APPENDIX 1.**

GUIDELINES FOR NEWCOMERS TO THE COLOFOL STUDY GROUP.

The COLOFOL STUDY GROUP is open to new local investigators. Inclusion of patients in the study will go on until the end of 2008.

Do you want to join the COLOFOL STUDY GROUP? Then this is what you should do:

1. Make CT scans according to the guidelines in appendix 1B-1D.

2. Fill in the Radiological CT protocol questionnaire (appendix 1E) and send the CTs and the questionnaire to Lennart Blomqvist or Dennis Tønner Nielsen.

3. Fill in the department registration form (appendix 1F) and send it to

   COLOFOL
   C/o Peer Wille-Jørgensen
   Department of Surgery K
   Bispebjerg Hospital
   Bispebjerg Bakke 23
   2400 København NV

When your CT scans have been approved, you will receive a login and a password to the COLOFOL database.

## Appendix 1 A

## COLOFOL

## Common side protocol
## Bank of blood and tissue samples

### Blood samples

Three samples of 10 ml peripheral blood are collected preoperatively prior to any neoadjuvant chemotherapy and/or radiotherapy. 10 ml is drawn in one EDTA tube, 10 ml in one heparinized tube, and 10 ml in one serum tube.

Samples are spinned for 15 minutes at 1500G within 30 minutes after collection.

The spinned EDTA tube and the spinned heparinized tube samples are split into 10 fractions: 3 x 1.6 ml plasma, 1 x 1.6 ml buffy coat, and 1 x 1.6 ml erytrocyte fraction.
The spinned plasma tube sample is split into 3 x 1.6 ml. Altogether 13 fractions collected in cryotubes are present after the separation.

The 13 cryotubes are immediately frozen at $-80^0$C until analysis.

### Tissue samples

If possible due to the tumour size, four biopsies each measuring appr. 0.5 x 0.5 x 0.5 cm are cut from the fresh, unfixed tumour immediately after the specimen has been resected. The biopsies are stored in cryotubes marked T1, T2, T3, T4 (tumour).
A biopsy of at least 0.5 x 0.5 cm is cut from the macroscopically normal mucosa appr. 10 cm from the tumour. The normal biopsy is put in a cryotube marked N (normal).
All tissue samples should be snapfrozen, and stored at $-80^0$C until analysis.

### Storage

Blood and serum samples are stored at each investigational center until analysis.
As a general agreement, all samples are available for COLOFOL research projects after approval of the COLOFOL Steering group.

**APPENDIX 1 B**

**COLOFOL**

Guidelines

Liver CT

Patient position: Supine
Oral contrast material: Water
Breath-hold: One single breath-hold in each phase in maximum inspiration

The values in the protocol are recommended parameters.

PROTOCOL

| | | AFTER CONTRAST | | |
|---|---|---|---|---|
| **PROTOCOL** | **BEFORE CONTRAST** | **SINGLE-SLICE** | **4 - SLICE** | **16 - SLICE** |
| | | | | |
| SCAN COVERAGE | liver | liver | liver | liver |
| COLLIMATION | | 5mm | 4x2,5 | 16x1,5 |
| PITCH | | | 1,25 | 1,5 |
| ROTATION TIME (sec) | | 1 | 0.75 | 0.75 |
| kV | same parameters as after contrast | 130 | 120 | 120 |
| mAs | | 180 | 180 | 200 |
| Matrix min/filter | | 512/B | 512/B | 512/B |
| **IV-kontrast** | | | | |
| Concentration | | 300 mg I / ml | 300 mg I / ml | 300 mg I / ml |
| Volume | | 2 ml / kg (max 180 ml) | 2 ml / kg (max 180 ml) | 2 ml / kg (max 180 ml) |
| Flow | | 4 ml/sec | 4 ml/sec | 4 ml/sec |
| Scan delay | | 50 sec | 65 sec | 70 sec |
| Preparation | 2 glasses of water | 2 glasses of water | 2 glasses of water | 2 glasses of water |

**Post-processing**

The images should be evaluated with different W/L settings. Especially specific liver window settings are recommended for evaluation.

After the baseline scanning all parameters have to be constant in subsequent control examinations.

**APPENDIX 1 C**

**COLOFOL**

**Guidelines**
**Liver and abdominal CT**

Patient position: Supine
Oral contrast material: Barium – or iodic contrast material (abdomen) and water (liver)
Breath-hold: One single breath-hold in each phase in maximum inspiration

The values in the protocol are recommended parameters.

**PROTOCOL**

| PROTOCOL | BEFORE CONTRAST | AFTER CONTRAST<br>**SINGLE-SLICE** | **4 - SLICE** | **16 - SLICE** |
|---|---|---|---|---|
| | | | | |
| SCAN COVERAGE | liver | Liver + abdomen | Liver + abdomen | Liver + abdomen |
| COLLIMATION | same parameters as after contrast | 5mm | 4x2,5 | 16x1,5 |
| PITCH | | | 1,25 | 1,5 |
| ROTATION TIME (sec) | | 1 | 0.75 | 0.75 |
| kV | | 130 | 120 | 120 |
| mAs | | 180 | 180 | 200 |
| Matrix/filter | | 512/B | 512/B | 512/B |
| **IV-kontrast** | | | | |
| Concentration | | 300 mg I / ml | 300 mg I / ml | 300 mg I / ml |
| Volume | | 2 ml / kg (max 180 ml) | 2 ml / kg (max 180 ml) | 2 ml / kg (max 180 ml) |
| Flow | | 4 ml/sec | 4 ml/sec | 4 ml/sec |
| Scan delay | | 50 sec | 65 sec | 70 sec |
| Preparation | 2 glasses of water | 2 glasses of water | 2 glasses of water | 2 glasses of water |

Post-processing

The images should be evaluated with different W/L settings. Especially specific liver window settings are recommended for evaluation.

After the baseline scanning all parameters have to be constant in subsequent control examinations.

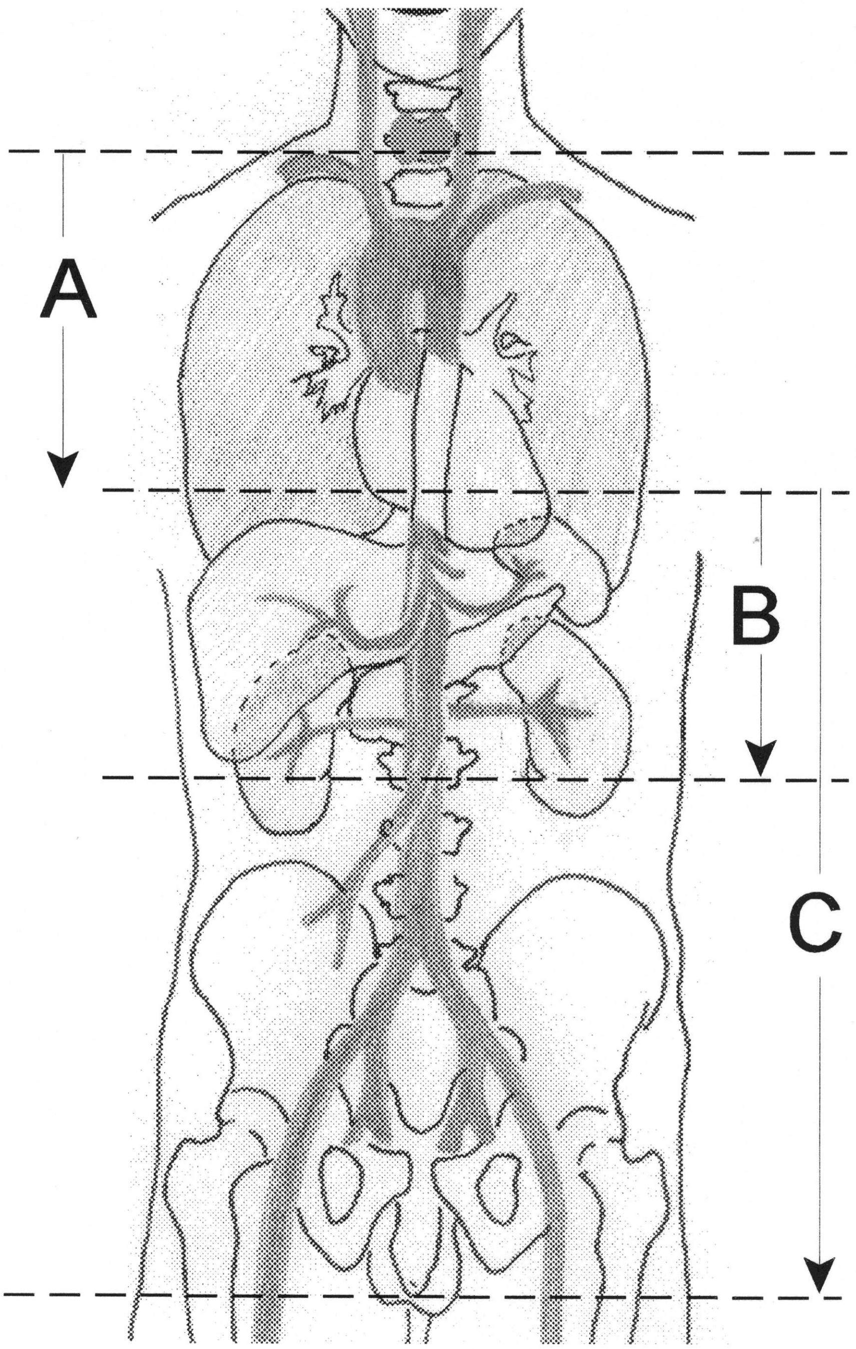


**A = Thorax**
**B = Liver**
**C = Liver/abdomen**

**APPENDIX 1 D**

**COLOFOL**

**Guidelines**

**CT-Chest – low dose**

Patient position: Supine
Oral contrast material: -
Breath-hold: One single breath-hold in each phase in maximum inspiration

The values in the protocol are recommended parameters.

**PROTOCOL**

| | **Single slice** | **4-slice** |
|---|---|---|
| Scan coverage: | Thorax | Thorax |
| Increment | 5 mm | 3.2 mm |
| Collimation | 5 mm | 4 x 5 mm |
| Pitch | 1.5 | 1.75 |
| Rotation time | 1.0 | 0.75 sec. |
| KV | 130 | 120 |
| mAs | 65 | 40 |
| Matrix/fil ter | 512/B | 512/B |
| IV contrast | | - |
| Concentration | | - |
| Volume | | - |
| Scan delay | | - |

After the baseline scanning all parameters have to be constant in subsequent control examinations.

**APPENDIX 1E**

## COLOFOL RADIOLOGICAL CT PROTOCOL QUESTIONNAIRE

**Hospital:........................................................ Address:...............................................**

**Surgeon..........................................................Address:................................................**

**Radiologist:....................................................E-mail:...................................................**

**Technologist:...................................................Email:....................................................**

**Phone:............................................................Fax:.......................................................**

**Equipment 1 (manufacturer/name/year):....................../............................./....................**

**Number of detectors:........................................................................................................**

**Equipment 2 (manufacturer/name/year):....................../............................/....................**

**Number of detectors:......................................................................................................**

**Equipment 3 (manufacturer/name/year):....................../............................/....................**

**Number of detectors:.......................................................................................................**

This questionnaire (three pages) is sent to (for Sweden):

Lennart Blomqvist M.D. Ph.D.
ADR Centrala röntgen
Karolinska Universitetssjukhuset Solna
171 76 Stockholm
Phone: 0046-8-51776117

(For Denmark and Poland)
Dennis Tønner Nielsen, Overlæge
Radiologisk afd. R
Århus Universitetshospital
Århus Sygehus
Nørrebrogade 44
8000 Århus
Denmark

For correspondence use also: **lennart.blomqvist@kirurgi.ki.se** or **dtnie@as.aaa.dk**

After recieving the questionnaire, the corresponding site (surgeon/radiologist/technologist) will receive a confirming e-mail.

**COLOFOL**

## Liver CT    RADIOLOGICAL PROTOCOL QUESTIONNAIRE

**Patient position:..........................................................................................................**

**Oral contrast material:.........................................................................................**

**Breath-hold:..........................................................................................................**

| PROTOCOL | BEFORE CONTRAST | AFTER CONTRAST Equipment 1 | Equipment 2 | Equipment 3 |
|---|---|---|---|---|
| | | | | |
| SCAN COVERAGE | liver | liver | liver | liver |
| COLLIMATION | | | | |
| PITCH | | | | |
| ROTATION TIME (sec) | | | | |
| KV | | | | |
| Mas | | | | |
| Matrix min/filter | | | | |
| IV-contrast | | | | |
| Concentration | | | | |
| Volume | | | | |
| Flow | | | | |
| Scan delay | | | | |
| Preparation | | | | |

## Liver and Abdominal CT

**Patient position:..........................................................................................................**

**Oral contrast material:.........................................................................................**

**Breath-hold:..........................................................................................................**

| PROTOCOL | BEFORE CONTRAST | AFTER CONTRAST Equipment 1 | Equipment 2 | Equipment 3 |
|---|---|---|---|---|
| | | | | |
| SCAN COVERAGE | liver | liver | liver | liver |
| COLLIMATION | | | | |
| PITCH | | | | |
| ROTATION TIME (sec) | | | | |
| KV | | | | |
| Mas | | | | |
| Matrix min/filter | | | | |
| IV-contrast | | | | |
| Concentration | | | | |
| Volume | | | | |
| Flow | | | | |
| Scan delay | | | | |
| Preparation | | | | |

**COLOFOL**

## CT THORAX  RADIOLOGICAL PROTOCOL QUESTIONNAIRE

Patient position:...........................................................................................................

Oral contrast material:.................................................................................................. -

Breath-hold:....................................................................................................................

**PROTOCOL**

| | Equipment 1 | Equipment 2 | Equipment 3 |
|---|---|---|---|
| Scan coverage: | | | |
| Increment | | | |
| Collimation | | | |
| Pitch | | | |
| Rotation time | | | |
| KV | | | |
| MAs | | | |
| Matrix/filter | | | |
| IV contrast | | - | |
| Concentration | | - | |
| Volume | | - | |
| Scan delay | | - | |

**Image storing** **PACS** أ

**Optical discs** أ

**DVD/CD** أ

**Film** أ

**General comments:**

## APPENDIX 1F

### REGISTRATION AS PARCIPATING DEPARTMENT IN COLOFOL

Department:.........................................................................................

Address...............................................................................................

...........................................................................................................

Country:............................................................

Name of local responsible investigator:.....................................................................

E-mail:.........................................................

Fax:.............................................................

Telephone:+.................................................

**Departments protocol ID:..............(to be filled out by COLOFOL secretariat)**

In order to participate in the COLOFOL study, you will have to fulfill following demands:

1.The National Ethical Comittee has accepted the protocol: Yes.... No.... Waiting........

2. I have read the full protocol and it´s appendices: Yes..... No.....

3. According to the protocol, I agree to:

a) include at least 50 patients during the study period, Yes….. No…….

b) include at least 30% of eligible patients Yes….. No …….

c) report on all included patients and their follow-up Yes …. No …….

d) report on all patients that fullfill the inclusion criteria eventhough they're hit by an exclusion criteria Yes….. No…….

e) inform the primary referring doctor about the project Yes…. No…….

f) inform the patient of his/her paticipation Yes ….. No……

4. I have made contracts with the local radiology department in order to have the planned investigations performed. Yes..... No..... Waiting.....

5. Participating in a local multidicplinary team in order to treat recurrences: Yes.... No.... Waiting.....

a) Name of oncology department:...................................

b) Name of Liversurgery department.................................

c) Name of Thoracicsurgery department................................

6. Being able to withhold local oncology departments from interferring with the COLOFOL-protocol. This means no extra investigations for recurrence unless symptoms Yes...... No......

7. I agree not to present any follow-up results of the allocated patients in the COLOFOL-study, orally and written, prior to the presentation of the COLOFOL-study. Yes.... No....

8: Being able to inform the primary referring doctor of the project: Yes... No....

9. Being able to inform the patient of his/her participation: Yes.... No.....

10. ……………………………………………………………………………………
Signature by the local responsible investigator

Send this form to:

COLOFOL
c/o Peer Wille-Jørgensen
Department of Surgery K
Bispebjerg Hospital
DK-2400 Copenhagen NV
DENMARK

Send CT-scans and CT questionnaire (see app. 1b-e) to:

Sweden and Uruguay:
Lennart Blomqvist
ADR Centrala röntgen
Karolinska Universitetssjukhuset Solna
171 76 Stockholm
Sweden

Denmark and Poland
Dennis Tønner Nielsen
Radiologisk afd. R
Århus Universitetshospital
Nørrebrogade 44
8000 Århus
Denmark

**For the COLOFOL secretariate exclusively:**

**Liver scans approved: Yes.... No.....**

**Department included: Yes.... No.….**

**Appendix 2**

# Algorithm for positive findings in follow-up of CRC according to the COLOFOL-protocol

## I. Serial CEA-measurements in follow-up of CRC

- CEA is tested 1 month postoperatively, for an individual baseline value.
- In further testing, the cut off level is defined as 5 pg/ml in individuals with baseline CEA levels < 5, while in individuals with baseline CEA levels >= 5, the cut off level is defined as 30% above baseline value.
- Further on CEA is tested at 12 and 36 months in the low frequency group and at 6, 12, 18, 24 and 36 months in the high frequency group, as part of the testpackage

**Algorithm for handling of CEA test results with negative scheduled CT-scan and negative CXR:**

1. If scheduled test is
   1.1. < 5 pg/ml in patients with base line value < 5, or
   1.2. < 30% above base line in patients with baseline value > 5.

   Patient will be followed as scheduled, according to protocol

2. If scheduled test is
   2.1. >= 5 pg/ml, but < 10 pg/ml in patients with base line value < 5 or
   2.2. >= 30% above base line but < 100% above base line in patients with base line value > 5

   A new test will be performed after 4 weeks.

   2.3. If this test shows a further increase, the test is regarded positive (see section II).
   2.4. If not, a new test will be performed after 4 weeks*

3. If scheduled test is
   3.1. >= 10 ng/ml, in patients with base line value < 5, or
   3.2. >= 100 % above base line value in patients with base line value > 5

   A new test will be performed immediately.

   3.3. If this test verifies the increase in CEA level, the test is regarded positive (see section II).
   3.4. If not, a new test will be performed after 4 weeks*

* The result will be handled according to the same principles as above for scheduled tests.

## II Algorithm for positive findings in follow-up

**1. Positive CEA test and negative scheduled abdominal CT**

1.1. CT thorax + MRI abdomen + pelvis and rigid sigmoidoscopy in rectal cancer

1.1.1. Positive finding: Evaluation in regional multidisciplinary board*
1.1.2. Negative finding (of complementary examinations): Evaluation in regional multidisciplinary board*. Options are: diagnostic laparoscopy, contrast enhanced ultrasonography, PET (if available) or new CT thorax + MRI of abdomen/CT in right side colon cancer (+ pelvis and sigmoidoscopy in rectal cancer) after 6 weeks

**2. Positive finding on scheduled CT thorax, abdomen or CXR** (+/- positive CEA)

2.1. CT thorax + MRI abdomen + pelvis and rigid sigmoidoscopy in rectal cancer

2.1.1. Positive finding: evaluation in regional multidisciplinary board*
2.1.2. Negative finding (of complementary examinations) with positive CEA; Evaluation in regional multidisciplinary board*. Options are: diagnostic laparoscopy, contrast enhanced ultrasonography, PET (if available) or new CT thorax + MRI of abdomen/CT in right side colon cancer (+ pelvis and sigmoidoscopy in rectal cancer) after 6 weeks
2.1.3. Negative finding (of complementary examinations) with negative CEA; follow-up according to protocol

## III Algorithm for positive findings in symptomatic patients

**1. "Malignant symptoms" or positive finding on any imaging for any reason**

1.1. CEA + CT thorax and MRI of abdomen + pelvis and rigid sigmoidoscopy in rectal cancer

1.1.1. Positive finding: evaluation in regional multidisciplinary board*
1.1.2. Negative finding (of complementary examinations) with positive CEA; Evaluation in regional multidisciplinary board*. Options are: diagnostic laparoscopy, contrast enhanced ultrasonography, MRI of abdomen/CT in right side colon cancer + pelvis and sigmoidoscopy in rectal cancer, PET (if available) or new CT thorax after 6 weeks
1.1.3. Negative finding (of complementary examinations) with negative CEA; follow-up according to protocol

**2. Bleeding per ani**

2.1. Sigmoidoscopy if rectal cancer; colonoscopy if colonic cancer

2.1.1. Positive finding: CT thorax + MRI of abdomen/CT in right side colon cancer + pelvis in rectal cancer and evaluation in regional multidisciplinary board*
2.1.2. Negative finding; follow-up according to protocol

*These boards also form the regional steering-groups for the study. They should preferably include surgeon, radiologist, and oncologist.

**Appendix 3**

**Guidelines for handling of detected recurrences in the COLOFOL study groups**

If recurrence is suspected or verified, the patient-case should be evaluated in a regional multidisciplinary board* to offer the best available treatment for the patient (surgery, palliative chemo and/or radiotherapy, no treatment).

In patients where a recurrence is diagnosed, a minimum requirement for treatment is recommended:

- Performance status of WHO 0 or 1 and ASA ≤3
- Sufficient liver and renal function
- Patient agrees to undergo surgery

Contraindications for curative aiming surgery are:

- Disseminated carcinomatosis (*e.g.* Virchow´s nodes or bone metastases)
- Multilobar lung metastases
- Performance status WHO >2 or ASA >4
- >6 liver segments involved or >75% tumour invasion of liver or all 3 hepatic veins involved (approximity to the liver veins is not a contraindication in RFA treatment)
- Major liver insufficiency or Child´s B or C liver cirrhosis with complications
- Patient declines surgery

As a general recommendation patients should be included in ongoing controlled clinical treatment studies that would not interfere with COLOFOL.

1. **Liver metastases**
   Patients with ≤4liver metastases where a liver resection preserves >30% of liver parenchyma should be resected. Whether neoadjuvant/adjuvant chemotherapy should be given is up to the regional multidisciplinary board to decide. The treatment when >4 metastases are present or when <30% of functional liver parenchyma would be preserved after radical aiming surgery is a decision by the regional board. The use of local ablation methods (radiofrequency/cryotherapy/laser) to achieve radical destruction of known lesions is allowed, but not recommended for lesions that can be radically resected.

2. **Lung metastases**:
   Patients with unifocal lung metastases without hilar nodes and not requiring pulmectomy should be resected or RFA treated in controlled clinical trials.

3. **Local recurrences**
   If a radical (R0 or R1) resection is judged possible at the multidisciplinary board discussion – a resection is favoured. This includes extensive surgery like: peritoneal resection, abdominal wall resection, hysterectomy, cystectomy and pelvic exenteration if necessary and if patient is fit. En-bloc resections are mandatory. Surgery can be combined with chemoradiation, including IORT, if judged beneficial to the patient.

4. **Multifocal recurrences**
   4.1. Synchronous liver and lung metastases:

Patients fulfilling the criteria for treatment of liver or lung metastases above should also be evaluated for resections/ablation when both liver and lung metastases are present.

4.2. Lymph node metastases and synchronous liver and/or lung metastases:
Radical aiming resections/ablations are not recommended as single treatment and should only be considered in combination with chemo/chemoradiation treatment performed in controlled clinical studies.

4.3. Local recurrence and synchronous liver and/or lung metastases:
Radical aiming resections/ablations are not recommended as single treatment and should only be considered in combination with chemo/chemoradiation treatment performed in controlled clinical studies.

*These boards also form the regional steering-groups for the study. They should preferably include surgeons, radiologists, and oncologists.

## Appendix 4

## Quality of life instruments

The effect of follow-up on health related quality of life will be assessed by three validated self-administered instruments, as used in other trials (Grunfeld et al. BMJ 1996;313:66-669)

- The British version of the SF-36 (Ware et al. Boston: Health Institute, New England Medical Center, 1993).
- The European Organisation for Research and Treatment of Cancer core quality of life questionnaire (EORTC QLQ-C30) (Aaronson et al. J Natl Cancer Instit 1993;85:365-376).
- And the hospital anxiety and depression scale (Zigmond and Snaith Acta Psychiatr Scand 1983;67:361-370).

**Appendix 5**

**Members of the COLOFOL study-group**

**Denmark**
Søren Laurberg, Surg. Dept., Århus Univ. Hospital
Henrik Toft Sørensen, Dept. Clin. Epid., Århus Univ. Hospital
Mogens Rørbæk Madsen, Surgical Dept. A, Herning Centr. Hospital
Henrik Harling, Surg. Dept. K, Bispebjerg Hospital
Peer Wille-Jørgensen, Surg. Dept. K, Bispebjerg Hospital
Peter Christian Rasmussen, Surg. Dept. Århus Univ. Hospital
Dennis Tønner Nielsen, Dept. Radiology, Århus Univ. Hospital
Mette Vinther Skriver, Århus
Per Vadgaard Andersen, Dept Surgery, Svenborg
Hans B Rahr, Dept Surgery A, Odense University Hospital
Knud Erik Jensen, Dept Surgery, Esbjerg
Erling Østergaard, Viborg Hospital
Per Jess, Dept Surgery, Hillerød Hospital
Per Gandrup, Dept Surgery A, Aalborg
Henrik Christensen, Dept. of Surgery L, Århus Hospital
Allan G. Pedersen, Dept. Of Surgery, Randers Hospital

**Sweden**
Nils Lundqvist, Surgical Dept. Norrtälje Hospital
Lars Påhlman, Surg. Dept. Uppsala Univ. Hospital
Peter Naredi, Dept. of Surg., Umeå Univ. Hospital
Birger Sandzen, Dept. of Surg., Umeå Univ. Hospital
Gudrun Lindmark, Surg. Unit., Helsingborg Lasarett
Ingvar Syk, Surg. Clinic, Univ. Hospital, Malmö
Kennet Smedh, Dept. of Surgery, Central Hospital, Västerås
Lennart Blomqvist, Dept. Radiology, Solna
Michael Goldinger, St. Görans Hospital, Stockholm
Anna Martling, Dept of Surgery, Karoliska Solna, Stockholm
Johan Ottoson, Dept. of Surgery, Central Hospital Kristianstad
Monika Svanfeldt, Gastrocentrum, Karolinska Huddinge, Stockholm
Stefan Dedorson, Surgical Clinic, Södertälje Hospital
Mats Bragmark, Dept. of Surgery, Danderyd Hospital
Jonas Bengtson, Dept. of Surgery, Sahlgrenska University Hospital, Göteborg
Helena Laurell, Dept. of Surgery, Mora Hospital
Yngve Raab, Dept. Of Surgery, Södersjukhuset, Stockholm
Christoffer Odensten, Dept. Of Surgery, Sunderby Hospital, Luleå

**Poland**
Jósef Kladny, Clinic of Gen. and Onco. Surgery, Pomeranian Medical University
Adam Dziki, Dept. of Surgery, Medical University of Lodz

**Uruguay**
Luis A. Carriquiry, Dept. of Surgery, Maciel Hospital, Montevideo

**UK**
Andrew Renehan, Inst. of Canc. Stud., Manchester

This group will be expanded according to the participation of centres.

## Appendix 6

## Database forms

| PRE-RANDOMISATION DATASHEET | |
|---|---|
| **PERIOPERATIVE FORM** | |
| PatientID.: | 1 |
| Last name: | |
| Initials: | |
| Gender: | Male Female |
| **INCLUSION CRITERIA** | |
| Birthday: | (dd-mm-yy) |
| **EXCLUSION CRITERIA** | |
| Clinical diagnosis of HNPCC or FAP: | - |
| Local excision for colorectal cancer: | - |
| Life expectancy less than 2 years: | - |
| Inability to comply with the control or intense follow-up programme: | - |
| Participates in other clinical trials interfering with the control programmes: | - |
| **GENERAL HEALTH** | |
| Ever diagnosed with one of the following diseases: | Diabetes |
| | AMI, hypertension or other heart diseases |
| | Pulmonary disease |
| | Multiple sclerosis |
| | Cerebrovascular disease |
| | Other major disease |
| - specify: | |
| **LIFE STYLE** | |
| Do patient smoke: | - |
| Do patient have a daily alcohol consumption: | - |
| **PERIOPERATIVE DIAGNOSTICS** | |
| Lung metastases (X-ray, CT, MR): | - |

| | |
|---|---|
| Liver metastases (CT, MR): | - |
| Rectal cancer: | - |
| Distance from anal verge to the lower tumour edge (<= 15 cm): | cm. |
| Initially judged inoperable (fixed tumor): | - |
| Preoperative radiation: | - |
| Preoperative chemotherapy: | - |
| **SURGERY** | |
| Date of operation: | (dd-mm-yy) |
| Elective or emergency operation: | - |
| Tumour perforation: | - |
| Tumour fixation: | - |
| Localization of colorectal cancer at operation: | ☐ Right side<br>☐ Transversum<br>☐ Left side<br>☐ Rectum (<=15 cm) |
| Temporary ostomy: | - |
| Permanent ostomy: | - |
| **Biological bank:** | |
| Blood sample to biological bank: | - |
| Sample tissue to biological bank: | - |
| **PATHOLOGY** | |
| Number of nodes examined (all patients): | |
| Number of nodes with metastases: | |
| Distance from tumour to the circumferential margin of resection (for rectal cancer the minimum distance): | mm |
| **Staging** | |
| - R0 (Macroscopic and microscopic local radicallity. No distant metastases.): | - |
| - Dukes' stage: | - |
| - TNM T-stage: | - |
| - TNM N-stage: | - |

| | |
|---|---|
| - TNM M-stage: | - |
| **POSTOPERATIVE COURSE** (<=30 days) | |
| Preoperative CEA level: | |
| Postoperative CEA level (4 weeks): | |
| Severe postoperative complications: | - |
| | ☐ Stroke |
| | ☐ Myocardial infarction or heart deficiency |
| | ☐ Pulmonary embolus |
| | ☐ Healing complications + laboratory |
| | ☐ Healing complications without laboratory |
| | ☐ Anastomosis + reoperation |
| | ☐ Anastomosis without reoperation |
| | ☐ Other serious events |
| - specify: | |
| Blood transfusion during the hospitalization: | - |
| Referred to postoperative radiotherapy: | - |
| Adjuvant chemotherapy planned: | - |
| Colonoscopy for clean colon or X-ray of Barium performed preoperatively or planned 3 months after surgery: | - |
| Signed consent for participation: | - |
| Date of filling in the form: | (dd-mm-yy) |

Submit Save Cancel

| FOLLOW-UP | |
|---|---|
| PatientID.: | 1 |
| Date of conclusion of this visit: | (dd-mm-yy) |
| Follow-up: | - |
| - If not planned, which cause/symptom: | ☐ Pulmonary symptoms |

| | |
|---|---|
| | ☐ Jaundice symptoms |
| | ☐ Gastrointestinal symptoms |
| | ☐ Pain |
| | ☐ Suspicious of local recurrence |
| | ☐ Other |
| - specify: | |
| **Examinations** (planned or interval) | |
| CEA: | - |
| - Reason if not performed: | |
| - CEA level: | |
| - level changed: | - |
| Multislice CT of liver / MR: | - |
| - Reason if not performed: | |
| CT of lungs / X-ray: | - |
| - Reason if not performed: | |
| Positive findings on any examinations above leading to further examinations: | - |
| | ☐ MR abd., MR pelvis |
| | ☐ CT thorax |
| | ☐ PET |
| | ☐ Repeated CEA |
| | ☐ Rigid sigmoid-oscopy |
| | ☐ Evaluation in multidisciplinary board |
| **Outcome** | |
| Recurrence of colorectal cancer denied: | - |
| Recurrence of colorectal cancer suspected: | - |
| Recurrence of colorectal cancer operable: | - |
| Location of metastasis: | - |
| Recurrence of colorectal cancer confirmed: | - |
| - How many: | |

| | |
|---|---|
| - Size of the largest (maximum): | mm |
| Metachronous cancer determined: | - |
| **Blood samples** | |
| Blood samples to biological bank: | - |
| Tissue samples to bio bank: | - |

Submit Save Cancel

## ENDPOINT / Termination

| | |
|---|---|
| PatientID: | 1 |
| **Endpoint** | |
| Reason for leaving: | - |
| - Death: | - |
| - Death related to examinations or complications: | - |
| | ☐ Examinations within the protocol |
| - specify: | |
| | ☐ Complications to secondary surgery |
| | ☐ Complications to radiation |
| | ☐ Complications to chemotherapy |
| Recurrence of colorectal cancer: | - |
| Date of leaving study: | (dd-mm-yy) |

Submit Save Cancel

**Appendix 7**

**Steering-group members:**

Peer Wille-Jørgensen, Bispebjerg Denmark
Søren Laurberg, Århus, Denmark
Dennis Tønner Nielsen, Århus, Denmark
Ingvar Syk, Malmö, Sweden
Kennet Smedh, Västmanland, Sweden
Adam Dziki, Lodz, Poland
Andrew Renehan, UK

**Appendix 8**

**Monitoring commitees**

**Study monitoring:**
To be appointed by Danish Colorectal Cancer Group
To be appointed by Swedish Colorectal Society
To be appointed from UK and the Netherlands

**Quality of diagnostic methods committee:**

Dennis Tønner Nielsen
Overlæge
Radiologisk afd. R
Århus Universitetshospital
Århus Sygehus
Nørrebrogade 44
8000 Århus , Århus
Denmark

Lennart Blomqvist M.D. Ph.D.
ADR Centrale röntgen
Karolinska Universitetssjukhuset, Solna
171 76 Stockholm,
Sweden

## Appendix 9

## Patient Information and Layperson protocol

### (For Denmark)

**Det videnskabelige projekt COLOFOL: "Hyppig kontrol efter operation for kræft i tyk- eller endetarm ?"**

Vi ved, at nogle patienter får tilbagefald af sygdommen, selvom alt kræftvæv tilsyneladen-de blev fjernet ved operationen. Det er derimod usikkert, om man ved hospitalskontrol kan opdage tilbagefaldet før patienten får nye symptomer, og på den måde forbedre helbredel-sesmulighederne. Derfor er der i øjeblikket ikke retningslinier for, om og hvordan en even-tuel kontrol efter operation bør tilrettelægges. De kirurgiske afdelinger har således meget forskellige kontrolprogrammer, og nogle slet ingen.

I de senere år er der kommet bedre skanningsmetoder til (især CT-skanning), og det er muligt, at disse metoder kan forbedre et kontrolprogram. Vi ved det ikke, og vi ved heller ikke hvor hyppigt vi skal kontrollere efter operation. Vi kan kun få et svar på det spørgsmål gennem et lodtrækningsforsøg: hyppig kontrol eller kontrol med lange mellemrum. Der er fordele og ulemper ved begge:

Fordelen ved hyppig kontrol er, at et tilbagefald muligvis kan opdages i tide, så helbredel-se er mulig . Ulemperne er, at nogle patienter bliver ængstelige ved udsigten til hyppige sygehusbesøg; af og til finder man noget ved undersøgelserne, der alligevel ikke viser sig at være kræft, og undersøgelsesmetoderne vil næppe kunne afsløre tilbagefaldet, hvis det er meget lille. Fordelen ved sjælden kontrol er, at man slipper for at blive mindet om den kræftsygdom, man er blevet opereret for. Ulempen er, at helbredelsesmulighederne muligvis ikke er gode, hvis man først behandles, når der er kommet symptomer på tilbagefald.

Vi spørger derfor, om du vil være med i vort forsøg. Du er velkommen til at tage dine pårørende med til samtalen, og vedlagt dette brev finder du pjecen: "Før du beslutter dig". Vi vil understrege, at din beslutning ikke får betydning for din behandling og kontrol. Hvis du siger ja, er det din fulde ret at trække dig ud igen på et hvilket som helst tidspunkt siden hen, uden det får indflydelse på din behandling. Hvis du siger nej, vil du blive tilbudt kontrol efter din afdelings normale procedure. Vi kan desværre ikke tilbyde dig hyppig kontrol uden for projektet – dette program er dyrt, vi ved ikke om det virker og må derfor prioritere vore ressourcer.

Der vil blive trukket lod om hyppig kontrol: 6,12, 18, 24 og 36 måneder efter operationen i form af CT-skanning eller røntgenundersøgelse af lungerne + CT-skanning af leveren + en blodprøve. I den anden gruppe med sjælden kontrol vil de samme undersøgelser blive foretaget 12 og 36 måneder efter operationen.

Ved hyppig CT-skanning vil du blive udsat for en forøget, men dog stadig ganske lille stråledosis (ca. 10 millisievert, hvilket svarer til to gange den bestråling, vi normalt udsættes for fra omgivelserne på et år). Risikoen for, at denne stråledosis fører til kræftudvikling er meget lille – mellem 1:3000 og 1:16.000. Risikoen for, at en dansker får kræft i løbet af sin levetid er til sammenligning 1:4.

Projektet kaldes COLOFOL; det er økonomisk støttet af Nordisk Cancer Union, ingen enkeltpersoner har økonomiske interesser i projektet, som foregår i flere europæiske lande.

Med venlig hilsen

| | |
|---|---|
| xxxxxxxx<br>overlæge<br>xxx - sygehus | Peer Wille-Jørgensen<br>Overlæge dr.med.<br>Projektleder<br>Bispebjerg Hospital |

Navn CPR (label)

Jeg bekræfter hermed at have modtaget skriftlig og mundtlig information om deltagelse i undersøgelse af værdien af efterkontrol efter behandling for tarmkræft.

Med min underskrift godkender jeg deltagelse i COLOFOL projektet.

xxxxxx den / 200

underskrift

Modtaget af læge:

Ikke-videnskabelig beskrivelse af COLOFOL projektet.

Værdien af kontrol efter operation for kræft i tyk-og endetarm er uafklaret, men de nyeste analyser af de videnskabelige resultater tyder på, at man ved mere intensiv kontrol kan opnå en forlænget overlevelse. Det ser desuden ud til, at det man skal lede efter er sygdomstilbagefald i leveren (levermetastaser), der er tilgængelig for behandling i en del tilfælde.

I øjeblikket er der ingen faste retningslinier for kontrol i Danmark og alle variationer af kontrolmetoder og intervaller anvendes på de forskellige sygehuse. Kontroller er dyre og kan medføre unødig ængstelse for patienterne, hvorfor en afklaring af de optimale kotrolintervaller og metoder er vigtig for både patienter og samfund.

Der er derfor planlagt et internationalt multicenterstudie hvor man ønsker afklaret hvorvidt et kontrolprogram, der indebærer scanning og/eller røntgenundersøgelse af lever og lunger, samt en blodprøve, der kan give mistanke om tilbagefald har nogen værdi.

Patienterne vil efter informeret samtykke ved lodtrækning blive placeret i to grupper. èn hvor kontrolprogrammet foretages efter 12 og 36 måneder og en anden, hvor programmet foretages efter 6, 12, 18, 24 og 36 måneder.

Såfremt der konstateres tilbagefald vil den videre behandling blive fastlagt af et tværfagligt panel bestående af kirurger, medicinske kræftlæger, og røntgenlæger.

Undersøgelsen opstartes som et multicenter studie i Sverige, Danmark, England og Holland.
De forberedende arbejder til undersøgelsen er støttet af Nordisk Cancerunion med 175.000 kr

Dansk version af COLOFOL protokol vedlægges.

København den 12/10 2004

Med venlig hilsen

Peer Wille-Jørgensen
Projektleder
Overlæge, dr.med.
Kirurgisk afdeling K
H:S Bispebjerg Hospital

**Patientinformation (for Sweden)**

# Patientinformation

Du tillfrågas härmed om Du vill delta i en forskningsstudie. Du har fått information om att vi har opererat bort en cancertumör i Din tjocktarm. Tumören har blivit radikalt bortopererad. Trots detta finns en risk att Din tumör kan ha spridit sig till olika organ. Inför operationen undersökte vi Dina lungor och lever och under operation har vi undersökt dessa organ också och kunnat konstatera att det inte finns någon spridning. Det kan dock finnas en mikroskopisk spridning, vilket innebär att det finns tumörceller som vi inte kan upptäcka idag. Skulle så vara fallet finns möjlighet att upptäcka dem genom ett kontrollerat uppföljningsprogram.

I Sverige kontrolleras patienterna regelbundet på mottagningen med ungefär 1 års mellanrum. Frågan har uppstått om man behöver kontrollera våra patienter ännu oftare och därför görs en stor studie i Sverige, där vi jämför uppföljning två gånger postoperativt (1 år och 3 år) med att följa våra patienter var 6:e månad. Denna studie görs tillsammans med kirurger inom de nordiska länderna, Holland och England. Vid varje uppföljningstillfälle kommer vi att ta blodprover, där vi specifikt tittar på leverfunktionen. Vi kommer att ta speciella tumörmarkörer samt ta till vara blodprov som skall sparas för att vi vid ett senare tillfälle skall kunna titta om specifika idag icke kända tumörmarkörer har blivit stegrade. Vid varje besökstillfälle kommer vi att ta 20 ml blod för dessa analyser. Hälften av dessa blodprover kommer att sparas i en s k biobank, vilket innebär att vi skall kunna gå tillbaka och titta på de specifika tumörmarkörerna. Vi kommer också att undersöka Din lever med datortomografi eller ultraljud samt undersöka Dina lungor. En undersökning med datortomografi motsvarar en stråldos på i storleksordningen 2,3 års bakgrundsbestrålning. Att undersöka just lever och lungor beror på att det i första hand är dit som en tumör brukar sprida sig.

I samband med den här undersökningen kommer vi att be Dig fylla i en enkät om hur Du upplever att bli undersökt på detta sätt för att om möjligt kunna hitta en tumörspridning som vi kan bota.

Personuppgiftsansvarig är Uppsala universitet. Enligt personuppgiftslagen (PUL) har Du rätt att gratis en gång per år få ta del av de uppgifter om Dig som hanteras och vid behov få eventuella fel rättade. Om Du vill ha detta utdrag, kontakta undertecknad, Lars Påhlman.

Det står Dig helt fritt att tacka nej. Du kan också när som helst tacka nej under den pågående studien utan att ange varför. Skulle Du avstå från att delta eller icke vilja fortsätta i studien, kommer detta inte att menligt påverka Dina möjligheter till ett gott omhändertagande.

Lars Påhlman
*Professor*
Kirurgiska kliniken, Akademiska sjukhuset, 751 85 Uppsala
E-post: lars.pahlman@surgsci.uu.se
Tel: 018-611 46 75

**Patientinformation (for Poland)**

Szanowna/y Pani/Panie

Chcielibyśmy Pani/Panu zaproponować uczestnictwo w projekcie badawczym **,,Pragmatyczne badanie nad oceną częstości badań diagnostycznych po resekcji jelita u pacjentów z II i III stopniem zaawansowania gruczolakoraka jelita grubego - badanie randomizowane, wieloośrodkowe."** zwanym dalej COLOFOL.

Projekt ten jest związany z Pani/Pana chorobą. Tak dla każdego człowieka przykra diagnoza jaką niedawno Pani/Pan usłyszeli wiąże się z wieloma działaniami jakie personel medyczny podejmuje wyłącznie w jednym, najważniejszym celu. Tym celem jest całkowite wyleczenie Pani/Pana z choroby nowotworowej jelita grubego. Najważniejszym elementem tego celu jest leczenie operacyjne, któremu już została/ł Pani/Pan poddana/y. Jeśli istnieje taka konieczność zgodnie z obowiązującymi standardami przy pewnym stopniu zaawansowania choroby nowotworowej należy też poddać pacjenta terapii dodatkowej przedoperacyjnej, bądź pooperacyjnej. Z kolej przychodzi czas na etap kolejny nie mniej ważny. Etapem tym jest okres obserwacji pacjenta po udanym leczeniu radykalnym. W tym czasie obserwuje się pacjenta i wykonuje pewne badania, aby sprawdzić czy nie nastąpił nawrót choroby. Jest to bardzo ważny okres, gdyż w razie wykrycia nawrotu choroby medycyna ma możliwości aby z tym walczyć i pozbyć się choroby.
Do tej pory nie ma obowiązujących wytycznych ani standardów jakie badania i jak często przeprowadzać aby wykrywanie nawrotu choroby było jak najszybsze, jak najefektywniejsze a jednocześnie nie utrudniało życia pacjenta. Istnieje wśród lekarzy duża zgodność co do tego jakie badania należy wykonywać. Są to:

- tomografia komputerowa jamy brzusznej, jest nowoczesnym badaniem z wykorzystaniem promieniowania rentgenowskiego
- badanie poziomu marker'a nowotworowego o nazwie antygen karcynombrionalny (CEA), jest związane z pobraniem niewielkiej ilości krwi
- zdjęcie rentgenowskie klatki piersiowej
- badanie ultrasonograficzne jamy brzusznej

Niestety nikt do tej pory nie określił jak często badania te powinno się wykonywać. Badanie, w którym proponujemy udział ma na celu określenie czy częstsze wykonywanie badań ma wpływ na długofalowe wyniki leczenia i jakość życia pacjenta.
Po wyrażeniu zgody na uczestnictwo w badaniu będzie Pani/Pan przydzielona/y do jednej z dwóch grup Obie gruby pacjentów będą objęte obserwacją pooperacyjną, w obu grupach będziemy wykonywać te same badania diagnostyczne a mianowicie tomografię komputerową jamy brzuszne, zdjęcie rentgenowskie klatki piersiowej, oznaczanie poziomu markera nowotworowego oraz

badanie ultrasonograficzne jamy brzusznej. Różnica będzie polegała na częstości wykonywania badań. a mianowicie

1. **schemat o mniejszej częstotliwości badań**

- CEA okołoperacyjnie i jeden miesiąc po operacji
- CEA, CT i rtg klatki piersiowej 12 i 36 miesięcy po leczeniu operacyjnym

2. **schemat o większej częstotliwości badań:**

- CEA okołooperacyjnie i jeden miesiąc po operacji
- CEA, CT i rtg klatki piersiowej 6, 12, 18 i 36 miesięcy po leczeniu operacyjnym.

Jak Pani/Pan widzi niezależnie od przydziału do gruby badawczej będą Państwo objęci skrupulatną i wnikliwą opieką pooperacyjna, której celem jest Pani/Pana pełne wyleczenie. Zapewniamy Pani/Panu anonimowość i pełną ochronę danych osobowych w razie przystąpienia do badania.
Zapewniamy, iż w każdej chwili bez podania powodu mogą Państwo zrezygnować z uczestnictwa w badaniu co nie będzie oznaczało, iż nie będziemy się państwem nadal opiekować z tą sama troska o Państwa zdrowie.
Zapewniamy, iż mimo nie wyrażenia zgody na uczestnictwo w badaniu będą Państwo objęci opieką naszej Kliniki.

Jednocześnie chcielibyśmy, aby Państwo pamiętali, iż wyrażenie zgody na uczestnictwo w badaniu będzie się wiązało z:

- koniecznością wizyt w Klinice (o różnej częstości w zależności od przynależności do różnej grupy badawczej),
- wykonaniu badań diagnostycznych (w tym z użyciem promieni rentgenowskich),
- możliwości kontaktowania się z państwem telefonicznie.

Postaramy się aby ewentualne uczestnictwo było dla Państwa jak najmniej kłopotliwe.

Z poważaniem

## Patientinformation in Spanish

INFORMACION AL PACIENTE

**Estudio científico “ COLOFOL”: el mejor intervalo entre los examenes de control luego de una operacion por cancer colorrectal – un estudio colaborativo entre cirujanos daneses, suecos, británicos, holandeses, polacos y uruguayos-**

Aunque la operación que le ha sido realizada ha sido exitosa y ha eliminado todos los tejidos afectados por el tumor, de acuerdo al criterio del cirujano, siempre existe un cierto riesgo de que la enfermedad reaparezca en el futuro. Existen en el mundo distintos programas de control que buscan despistar tempranamente la reaparición de la enfermedad para poder tratarla adecuadamente y a tiempo.
Pero no sabemos aun con que frecuencia y qué estudios son los más efectivos para lograr ese objetivo. No tendría sentido realizar estudios cada poco tiempo dado los gastos y las molestias que ello podría originar al paciente.
Por ello un grupo de investigadores europeos ha diseñado el presente estudio que busca establecer cual es el mejor de dos esquemas de seguimiento con respecto al mejor futuro para el paciente y con el menor gasto para la sociedad.
Un grupo de médicos uruguayos hemos solicitado participar en este estudio y nuestro pedido ha sido aceptado, por lo que incluiremos nuestros pacientes al mismo tiempo que los suecos, daneses, holandeses, polacos e ingleses.
Por eso le pedimos que acepte participar en este estudio. Decida participar o no , Ud. será tratado y controlado de acuerdo a los criterios de su cirujano y su oncólogo. Pero si decide hacerlo, Ud. será asignado luego de la intervención quirúrgica a uno de los dos programas de seguimiento y deberá cumplir con los estudios, que le solicite su cirujano, en los plazos fijados.
Los estudios a realizar en cualquiera de los dos programas son similares: estudios de sangre y tomografías o resonancias magnéticas, variando solamente los plazos en los que se realizarán. Estos estudios son los que habitualmente se realizan en nuestro país para todos los pacientes operados de cáncer colorrectal como Ud. Aceptando participar en el estudio, Ud. se compromete sólo a realizarse esos estudios en los plazos que le correspondan de acuerdo al programa que le sea asignado, sea cada a los 6, 12, 18, 24 y 36 meses durante 3 años, o solo a los 12 y 36 meses en el mismo plazo. En cualquiera de los dos casos, la dosis de radiación que implica la realización de tomografias computadas es mínima y no ofrece riesgos para su futuro. Si Ud. no acepta participar o desiste de hacerlo en cualquier momento luego de haber aceptado, se le seguirán realizando estudios similares a criterio de su cirujano u oncólogo, sin que se resienta por ello la calidad de su atención médica.
Este estudio no persigue otro fin que determinar con precisión científica cual es el mejor esquema de seguimiento para los pacientes operados con éxito por cáncer colorrectal, como es su caso. Deseamos dejarle claro finalmente:

- que este estudio sólo persigue un interés científico y que ninguna persona o institución tiene ningún tipo de interés económico en su realización.
- que su participación no lo hace acreedor a ningún tipo de retribución personal , salvo eventualmente un viático de transporte para aquellos pacientes que vivan en el Interior del país.
- que sus datos personales serán resguardados bajo secreto profesional y adecuadamente codificados en una base de datos central en Dinamarca

Esperamos su consentimiento. Ante cualquier duda no vacile en comunicarse con el Dr. Luis A. Carriquiry, investigador principal del proyecto en Uruguay ( tel 099 610926)

Otorgo mi consentimiento

Firma................................................ Nombre.............................................................

No otorgo mi consentimiento

Firma................................................. Nombre .............................................................

## Patientinformation and layperson protocol in English (translated from the Danish Version)

**The scientific investigation COLOFOL: The Interval beween control-examinations after operation for colorectal cancer.**

We know some patients will experience recurrent disease, despite all malignant tissue apparently were remoed at the operation. It is although uncertain whether you by means of hospital control investigations can diagnose these recurrencies before symptoms occur thus improving the chance of cure. The guidelines for which kind of control programmes to apply after the operation are thus variable from hospital to hospital.

During the later years better scanning methods have become available, and it is possible, that these methods can improve the control programmes. We do not know and we also do not know how often the controls are to be performed. We can only get an answer on this though a randomised study where the interval between controls are descided by the draw of a lot.

The advantage by a frequent control is that a recurrence eventually can be discovered in such a due time that cure is possible. The disadvantage is that some patients dislike frequent visits to hospitals, and sometime you find something which finally shoes up to be non recurrent disease. The control investigations might not find the recurrence if it is very small. The advantage by a less frequent control is that you are not reminded of the malignant disease, you have been operated for. The disadvantage is that the possibilities for cure might be less if treatment is postponed until symptoms occur.

We therefore ask whether you will participate in our trial. You are welcome to bring consult relatives and as an addition to this letter, you find the brochure “Before I Decide”. We want to emphasize, that your decision will have no influence on your treatment and control. If you accept, it is your right to withdraw your consent at any time without this will have any influence on your treatment. If you say no, you will be offered control according to the normal procedures in the department. We are not able to offer you frequent control outside the trial. This programme is expensive, and we do not know whether it works, thus we have give priority according to our resources

A lot wil be drawn in frequent control: 6, 12, 18, 14, 36 months after the operation a CT-scan or X-ray of the lungs, a CT-scan of the abdomen, and a blood sample will be performed. In the group of infrequent control the same investigations will be performed at 12 and 36 months after the operation

By frequent CT-scan you will be exposed to an elevated, but still very small dose of radiation (about 10 millisievert, which is double the dose, you normally gets from the environment per year). The risk this dose should lead to cancer is betwen 1:3000 to 1:16,000. The risk an average person develops cancer during his/her lifetime is about 1:4.

The trial is called COLOFOL and has been supported by the Nordic Cancer Union. No individual persons or companies have any economic interests in the trial, which is conducted in several European Countries.

Sincerely

xxxxxxxxxx
Consultant surgeon

xxxxxxxxxxxxxxx
Country co-ordinator, COLOFOL

**Non-scientific description of the COLOFOL trial (layman protocol)**

The value of control after operation for colorectal cancer is mostly unknown, but the newest analyses of the scientific results indicate that a more intensive control leads to a better survival. Besides this there are indications of that the thing to look for is recurrence in the liver (liver metastases) which is sometimes cureable.

The guidelines for control in Europe vary from hospital to hospital. Controls are expensive and can lead to unnecessary anxiety for the patient. It is thus important to clarify the optimal control intervals and methods for both patients and society

Therefore an international multicenter trial has been planned in which we want to clarify whether a control programme including scanning and/or X-ray of lungs and liver + a blood test, which can indicate recurrence are of any value.

After informed consent has been obtained the patients will after drawing a lot be placed in one of two groups. One where the programme will be run after 12 and 36 months or another where the programme will be run at 6, 12, 18, 24, and 36 months after surgery.

If recurrence is discovered the further treatment will be decided by a multidisciplinary team consisting of surgeons, oncologists, and radiologists.

The trial will performed in Denmark, Sweden, Poland and eventually UK and Holland. The planning of the study has been supported by the Nordic Cancer Union by 25,000 EUROS

**Appendix 10**

**Internet randomisation**

The randomisation will take place over the Internet via a server placed at Aarhus University Hospital, Denmark. The server contains a randomisation programme, which each centre will gain access to via a username and password. When logging on to the randomisation site, each centre must key patient data on all patients, who have given their consent and who fulfil the inclusion criteria for the study. After keying the data, a randomisation code will be provided.
The data is encrypted before the information is transmitted over the Internet.

**The project's database**

A database for the project will be established, and this will also be placed at Aarhus University Hospital, Denmark. The Danish Board of Registry has approved the database. Access to the database can be gained through the project's homepage, by providing a valid username and password. Keying of baseline- and follow-up data will be carried out over the Internet using a standard web-browser, so no programmes need to be installed on the computers located at the centre. A number of validation procedures will be installed in order to ensure a high data quality. There will be sent out reminders of all follow-up visits and examinations, and data from these will also be keyed via the Internet. Each centre will be able to log on to the database via the homepage at any time in order to see descriptive data and number of included patients for own centre as well as for the entire study population. The database will ensure that data is available for statistical analysis immediately after termination of the study.

**Appendix 11**

**Approval from Ethical Commitees**

**KOMISJA BIOETYKI UNIWERSYTETU MEDYCZNEGO W ŁODZI**
**Al. Kościuszki 4, 90-419 Łódź, tel. / fax. 6321485**
**Wydział Wojskowo – Lekarski, ul. Żeligowskiego 7/9, 90-752 Łódź, tel./fax. 6324087**

**UCHWAŁA KOMISJI BIOETYKI**
**O PROJEKCIE EKSPERYMENTU MEDYCZNEGO**
**numer RNN/185/05/KB z dnia 10.05.2005 r.**
(przy korespondencji dotyczącej niniejszej decyzji prosimy powoływać się każdorazowo na powyższy numer i datę **Uchwały**)

**Wnioskodawca badania:**
**Prof.dr hab.med. Adam Dziki,** kierownik Kliniki Chirurgii Ogólnej i Kolorektalnej Uniwersytetu Medycznego w Łodzi

**Tytuł badania:**
**„Pragmatyczne badanie nad oceną częstości badań diagnostycznych po resekcji jelita u pacjentów z II i III stopniem zaawansowania gruczolakoraka jelita grubego – badania randomizowane wieloośrodkowe"**

Na podstawie art. 29 ustawy z dnia 5 grudnia 1996 r. o zawodzie lekarza (Dz.U. z 1997 r. Nr 28 poz.152 wraz z późniejszymi zmianami), zarządzenia Ministra Zdrowia i Opieki Społecznej z dnia 11 maja 1999 r. w sprawie szczegółowych zasad powoływania i finansowania oraz trybu działania komisji bioetycznych (Dz.U.Nr 47 poz. 480), ustawy o prawie farmaceutycznym z dnia 20 kwietnia 2004 r. (Dz.U.Nr 92 poz. 882) oraz Zarządzenia Nr 13 Rektora Uniwersytetu Medycznego w Łodzi z 18 października 2002 r. wraz z Aneksem nr 1 z dnia 31 maja 2004 r., **Komisja Bioetyki Uniwersytetu Medycznego w Łodzi (wypełniając zobowiązania ICH GCP)** na posiedzeniu w dniu 10.05.2005 r. przeanalizowała wniosek, wysłuchała opinii recenzenta o przedstawionym projekcie badania i w wyniku przeprowadzonej dyskusji oraz tajnego głosowania

**podjęła uchwałę o**
**pozytywnym zaopiniowaniu tego wniosku**

**bez zastrzeżeń**

Do Komisji wpłynęły następujące dokumenty:
- Wniosek badacza
- Plan pracy
- Informacja dla pacjenta
- Formularz świadomej zgody pacjenta

Przewodniczący
Komisji Bioetyki Uniwersytetu Medycznego
w Łodzi

Prof.dr hab.med. Przedzisław Polakowski

**DE VIDENSKABSETISKE KOMITÉER FOR**
**KØBENHAVNS OG FREDERIKSBERG KOMMUNER**

Dato 27. december 2004

☐ Komité 1 ☐ Komité 2 J.nr. (KF) 01-194/04

Overlæge dr. med. Peer Anders Wille-Jørgensen
H:S Bispebjerg Hospital
Kirurgisk afd. K
Bispebjerg Bakke 23
2400 København NV

Vedrørende projektet (KF) 01-194/04: "Kontrol efter operation for kolorektal cancer. Et randomiseret forsøg af to kontrolregimer".

De Videnskabsetiske Komitéer for Københavns og Frederiksberg Kommuner har d. d. bedømt ovennævnte projekt som værende i overensstemmelse med Lov om de videnskabsetiske komitéer.

Det henstilles, at De selv underretter de øvrige medlemmer af projektgruppen om bedømmelsen.

Komitéen skal anbefale, at komitéens journalnummer så vidt muligt påføres de skriftlige forsøgspersoninformationer m.v. og anføres i forbindelse med publicering af projektet.

Med venlig hilsen

Rikard Vrogaard

/Lisbeth Lundgren

Fælles sekretariat: Københavns Kommune, Sundhedsforvaltningen, Sjællandsgade 40, 2200 København N
Telefon: 35 30 34 02, 35 30 34 05, 35 30 34 07, 35 30 34 09
Telefax: 35 30 39 90

EPN
REGIONALA ETIKPRÖVNINGSNÄMNDEN • UPPSALA •

**BESLUT**
**2005-02-01**

**Dnr 2004: M-453**

**SÖKANDE FORSKNINGSHUVUDMAN**

Uppsala Universitet
Box 256
751 05 Uppsala

**Forskare som genomför projektet:**

Lars Påhlman
Kirurgiska kliniken
Akademiska sjukhuset
751 85 Uppsala

---

**UPPGIFTER OM FORSKNINGSPROJEKTET ENLIGT ANSÖKAN INKOMMEN TILL NÄMNDEN 2004-11-23 SAMT INKOMMEN KOMPLETTERING 2005-01-31.**

**Projektbeskrivning**

Uppföljning av colorektalcancer - en randomiserad studie.

---

Regionala etikprövningsnämnden i Uppsala meddelar följande


| Adress | Telefon | Fax | E-post |
|---|---|---|---|
| Box 569<br>751 22 Uppsala | 018-4717400 | 018-4717410 | registrator@uppsala.epn.se |




**Dnr 2004-453**

**BESLUT**

Nämnden bifaller ansökningen med inkommen komplettering 2005-01-31 och godkänner med stöd av 6 § lagen (2003:460) om etikprövning av forskning som avser människor den forskning som anges i ansökan.

**Villkor:**
**Texten om personuppgiftslagen skall kompletterar med uppgift om kontaktperson.**
**Forskningsprojektet innebär att blodprover sparas i biobank. Nämnden erinrar om skyldigheten att inhämta s.k. informerat samtycke från provgivarna enligt 3 kap.1§ lagen om biobanker inom hälso-och sjukvården m.m. samt även vad som i övrigt följer av reglerna i nämnda lag.**

**Erinran**
Godkännandet upphör att gälla om forskningen inte har påbörjats inom två år efter slutgiltigt beslut.

**BESLUTET FÅR ÖVERKLAGAS**
Se bifogad anvisning.

På nämndens vägnar

Jerry Eriksson
Ordförande

Brita Karlström
Vetenskaplig sekreterare

**Exp. till:**
Forskare: Lars Påhlman
Forskningshuvudmannens behörige företrädare: stf.prefekt Olle Nilsson, Institutet för kirurgiska vetenskaper, Akademiska sjukhuset, 751 85 Uppsala

EL CONSEJO DE LA FACULTAD DE MEDICINA DE LA UNIVERSIDAD DE LA REPÚBLICA EN SESIÓN ORDINARIA DE FECHA 22 DE NOVIEMBRE DE 2006, ADOPTO LA SIGUIENTE RESOLUCIÓN:

49.

(Exp. N° 071140-001530-06) - Tomar conocimiento de que el Comité de Ética que estudia los Proyectos de Investigación aprobó el Proyecto titulado: "**COLOFOL - un estudio pragmático para determinar la frecuencia de las pruebas diagnósticas de seguimiento en pacientes con cáncer colorrectal estadios II y III sometidos a resección curativa**" cuyo investigador responsable es el Prof.Dr. Luis Carriquiry.-
(9 en 9)

Comuníquese al investigador y pase a la Comité de Ética para Proyectos (Depto. de Secretaría).

ES COPIA FIEL

GRISEL YARDINO
ADM. I

**Appendix 12**

**Flow-chart**

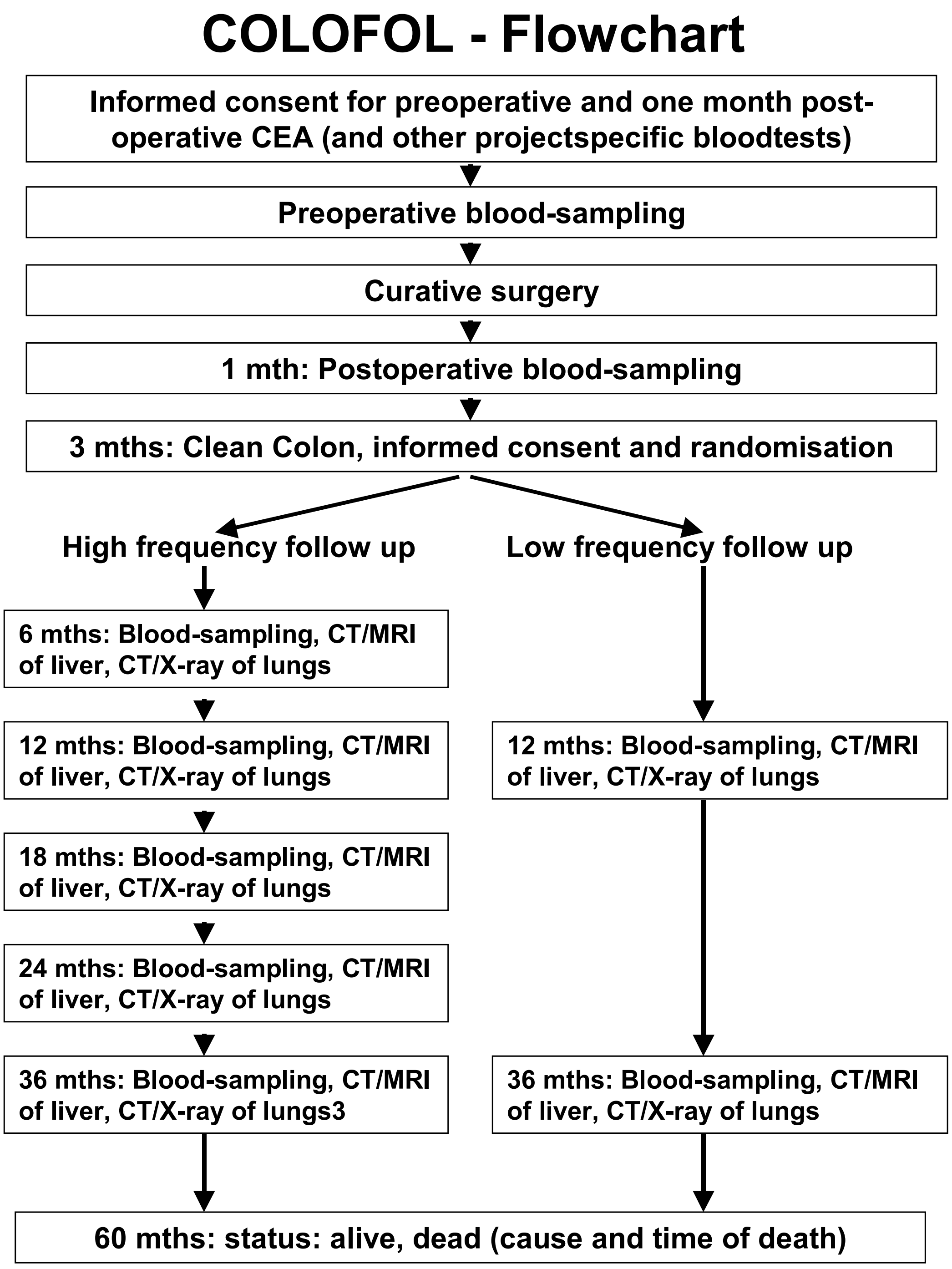

# COLOFOL – Inclusion and exclusion

Inclusion criteria:

- Radical surgery (R0-resection) for colorectal adeno-carcinoma – with or without adjuvant treatment, and
- Age $\leq$ 75 years, and
- Written informed consent, and
- "Clean colon" verified by perioperative barium enema or colonoscopy last 3 months post-surgery, and
- Tumour stage:II-III ($T_{any}$ $N_{1-2}$ $M_0$, $T_{3-4}N_{any}M_0$ ,Dukes´ B - C)

Exclusion criteria:

- A clinical diagnosis of HNPCC (non hereditary polyposis colorectal cancer) or FAP (familial polyposis coli),
- Local resection for colorectal cancer (*e.g.*, TEM-procedure),
- Life-expectancy less than 2 years due to concurrent disease (*e.g.*, cardiac disease, liver cirrhosis),
- Inability to provide informed consent or refusal to do so,
- Inability to comply with the follow-up programme,
- Participation in other clinical trials interfering with the control-programmes – *e.g.* TRIALS on adjuvant therapy with CT endpoints
- Other or previous cancer (except for non-melanoma skin cancer)

Pre – or postoperative adjuvant chemotherapy and/or radiation therapy is allowed, as long as it is applied with same indications in the two groups.

Endoscopy is permitted, as long as equal in the two groups.

# 2. eStatistical Analysis Plan

**AIM**

To conduct a prospective multicenter randomised study comparing total mortality, cancer-specific mortality, recurrence-free survival, economic cost effectiveness, and quality of life (QOL) in patients receiving high-volume follow-up after radical resection for colorectal cancer, compared to patients receiving low-volume follow-up.

**STUDY PROCEDURES**

*Recruitment and Eligibility*

At each participating center, consecutive patients receiving radical surgery for colorectal cancer will be considered for inclusion. Those who are ineligible or who refuse consent will be followed observationally.

*Inclusion criteria*

- Radical surgery (resection) for colorectal adenocarcinoma – with or without adjuvant treatment
- Age less than 75 years
- Provision of written informed consent for participation.
- "Clean colon" verified by perioperative barium enema or colonoscopy performed 3 months post surgery, at the latest
- Tumor stage: T2-T4, Nany, M0 or Dukes´ type B or C
- Pre- or postoperative chemotherapy and/or radiation therapy allowed.

*Exclusion criteria*

- Clinical diagnosis of HNPCC or FAP
- Local resection for colorectal cancer (*e.g.*, TEM procedure)
- Life expectancy less than 2 years due to concurrent disease (*e.g.*, cardiac disease, terminal multiple sclerosis, liver cirrhosis)
- Inability to provide informed consent or refusal to do so.
- Inability to comply with the control or intense follow-up programme.
- Participation in other clinical trials interfering with the follow-up programmes

*Informed consen*t (written) will be obtained within 30 days after surgery and after the primary tumor classification has been made. Patients refusing to participate will be asked for consent to be followed in an observational analysis, using data on their actual follow-up program and their clinical

course.

*Randomisation and treatments*

Randomisation will take place by telephone, E-mail (or FAX) from a central randomisation unit located in the offices of the Cochrane Colorectal Cancer Group. Randomisation will be stratified according to tumor stage and clinical centre. Randomisation will be blocked, in variable groups of 4 or 6. The allocation procedure will be concealed to participating care providers.

*Study Follow-up Regimens*

CEA will be measured on all colorectal cancer patients before surgery or preoperative adjuvant therapy and after the completion of primary treatment. All patients will have a “clean colon” within 3 months perioperatively, and at least one perioperative imaging procedure (Ultrasound, MR, or CT) of the liver and x-ray of the lungs. In both study groups an unlimited number of endoscopies will be allowed. Interval diagnostic evaluation also will be allowed for all subjects presenting symptoms.

1. *Low-volume follow-up regimen*
    - CEA, mulitislice CT/ or MRI of the liver and X-ray or CT of the lungs one year after surgery. For rectal cancer with re-established luminal continuity, an MR scan of the pelvis and/or endorectal ultrasound (by a dedicated sonographer) at 6, 12, 18, 24, and 36 months after surgery. For patients with abdominoperineal resection, an MR scan of the pelvis at the same intervals.
    - Patients will be instructed to contact their recruitment centre if symptoms of recurrence occur.
    - If recurrent disease is suspected, diagnostic measures and treatment used normally in the individual centre will be applied.
2. *High-volume follow-up regimen*
    - CEA, multislice hepatic CT or MR and/or PET scans at 6, 12, 18, 24, and 36 months. For rectal cancer with re-established luminal continuity, MR scan of the pelvis and/or endorectal ultrasound (by a dedicated sonographer) at 6, 12, 18, 24, and 36 months after surgery. Biopsies should be taken if suspected areas are found. For patients with abdominoperineal resection, MR scan of the pelvis at same intervals.
    - Patients will be instructed to contact their reference centre if symptoms of recurrence occur.
    - If recurrent disease is suspected, diagnostic measures and treatment used normally in the individual centre will be applied.

*Data to be obtained*: the following data will be obtained on all randomised subjects and those who give consent for observational study:

- Baseline demographic, clinical, and lifestyle factors (date of birth, major chronic illnesses, smoking, alcohol intake, medications, etc).
- Results of all diagnostic evaluations for metastatic disease during the initial evaluation
- Detailed description of initial surgical, adjuvant, and radiation treatment
- Findings from all cancer surveillance tests obtained after diagnosis
- Detailed description of all cancer treatments administered after randomisation
- Quality of life (QOL) assessment by telephone interview at 1, 3 and 5 years after randomisation. A standardised and validated QOL instrument, that takes follow-up regimens into consideration, will be used.

*Statistical analysis and power*

The primary effect parameter of the study will be total mortality and cancer-specific mortality after 5 years; the secondary endpoint will be time to diagnosis of recurrence (*i.e.*, recurrence-free survival) and quality of life.

Results will be evaluated both on an intention-to-treat basis and on an as-treated (per protocol) basis. Patients who withdraw their informed consent and thus change their follow-up programme will remain in their allocation group in evaluations conducted on an intention-to-treat basis, and will be excluded in evaluations conducted on a per-protocol basis.

Non-randomised patients and patients who withdraw their informed consent will be analysed separately.

On the basis of the results listed in Figure 1 in the protocol, an estimate of 5 years' mortality at 60% and a MIREDIF (Minimal Relevant Difference) of 6% seem justified. With a risk of type 1 error at 5% and type 2 error of 15%, about 1,100 patients should be randomised to each group. With an expected dropout rate of about 20%, the planned number of randomised patients should be 2,500. Survival data will be analysed according to the Kaplan-Meier method. Comparisons between groups will be made using the log-rank method. Binominal data will be analysed with Chi-square statistics and continuous data will be analysed with the Mann-Whitney U test.

Level of significance = 5%.

# Effect of More versus Less Frequent Follow-Up Testing on 10-Year Overall and Colorectal Cancer-Specific Mortality in Patients with Stage II or III Colorectal Cancer: a Post-trial Analysis of the COLOFOL Trial

## Statistical Analysis Plan

### *1) Study design*

The COLOFOL study group was established in 2004. Twenty-four recruitment centers in Denmark, Sweden, and Uruguay contributed patients to the trial. The objective of this prospective multicenter randomized trial was to examine overall and colorectal cancer-specific mortality rates in patients with Stage II or Stage III colorectal cancer who were randomized after undergoing curative surgery to 2 groups with alternative schedules for follow-up testing with computerized tomography (CT) scans and carcinoembryonic antigen (CEA) testing.

All centers participating in the COLOFOL trial were required by the protocol to follow up on all study participants with surveillance examinations until 3 years post-surgery and to report outcomes to the study's coordinating center until 5 years after surgery.

Although the trial did not yield entirely conclusive results, the findings revealed a small but potentially important clinical difference between the 2 groups.

This post-trial follow-up study aims to conduct a follow-up analysis of the patients included in the COLOFOL study in Denmark and Sweden that extends beyond the initial 5 years of follow-up.

### *2) Principles of analysis*

Results will be evaluated both on an intention-to-treat and an as-treated (per-protocol) basis. Patients who withdrew their informed consent and thus changed their follow-up regimen will remain in their allocation groups for the intention-to-treat analysis but will be excluded from the per-protocol analysis.

Patients who incurred a protocol violation during the study period (including participation in other clinical trials that interfered with the follow-up regimen), who developed a new primary cancer, or whose records are missing data on key variables will also be excluded from the per-protocol analysis.

### *3) Exposure variables*

Trial participants' data will be analyzed according to the group to which they were randomly assigned:

*Low-frequency follow-up regimen*

- CEA screening preoperatively and 1 month postoperatively. Missing preoperative CEA results will not be considered an exclusion criterion and will be handled as missing data.
- CEA screening, multi-slice CT or magnetic resonance imaging (MRI) of the liver, and X-ray/CT of the lungs at 12 and 36 months post-surgery. Patients were instructed to contact their recruitment center if they experienced any symptoms of disease recurrence.
- If recurrent disease is suspected, participants will undergo a standard work-up for diagnosis and treatment.

*High-frequency follow-up regimen*

- CEA screening preoperatively and 1 month postoperatively. Missing preoperative CEA results will not be considered an exclusion criterion and will be handled as missing data.
- CEA screening, multi-slice hepatic CT or MRI of the liver, and X-ray or CT of the lungs at 6, 12, 18, 24, and 36 months post-surgery. Patients were instructed to contact their recruitment center if they experienced any symptoms of disease recurrence.
- If recurrent disease is suspected, participants will undergo a standardized work-up for diagnosis and treatment (see Appendices 2–3).

### *4) Endpoints*

The following endpoints will be analyzed:

- 10-year overall mortality rates and
- 10-year colorectal cancer-specific mortality rates.

The outcomes will be obtained from population-based cancer, death, pathology, and patient registries in Denmark and Sweden. We will exclude patients from Uruguay because we are unable to obtain adequate follow-up information from these patients.

### *5) Analysis of baseline data*

Trial participants will be described according to the group to which they were randomly assigned and by categories of baseline characteristics. Categorical data will be presented as counts and percentages, and continuous data will be presented as medians and interquartile ranges.

### *6) Analysis of follow-up data*

Trial participants will be followed from the date of radical surgery for colorectal cancer until the date of the analyzed study outcome, date of dropout, date when lost to follow-up, or at 10-year follow-up, whichever comes first.

The Kaplan-Meier method and the log-rank test for between-group comparisons will be used to analyze 10-year overall mortality in the post-trial group participants. The colorectal cancer-specific mortality rates will be calculated using a cumulative incidence function and treating death associated with other causes as competing events. Risk differences in the 10-year overall and colorectal cancer-specific mortality will be calculated with corresponding 95% confidence intervals.

The main analyses will be stratified by cancer stage and will be repeated for the subgroups of patients diagnosed with rectal and colon cancer.

In the first sensitivity analysis, we will repeat the main analyses for trial participants from Denmark and Sweden. The overall and colorectal cancer-specific mortality rates will be compared between the 2 countries.

In a second sensitivity analysis, Cox proportional hazards regression will be used to compute crude and adjusted hazard ratios for overall and colorectal cancer-specific mortality rates. The hazard ratios will be adjusted for age at the time of colorectal cancer surgery; sex; colorectal cancer type; cancer stage; comorbidities including diabetes, cardiovascular disease, pulmonary disease, and cerebrovascular disease; preoperative radiation; adjuvant chemotherapy; and smoking.

The level of significance will be $P < .05$ with a 2-sided test. Statistical software package SAS (SAS Institute, Inc., Cary, NC, USA) will be used for data management and analyses.
