# Supplementary material for: More vs Less Frequent Follow-Up Testing and 10-Year Mortality in Patients With Stage II or III Colorectal Cancer: Secondary Analysis of the COLOFOL Randomized Clinical Trial
Source: JAMA Netw Open. 2024 Nov 21;7(11):e2446243. doi: 10.1001/jamanetworkopen.2024.46243 (PMC11582930; doi:10.1001/jamanetworkopen.2024.46243)
Supplement: Supplement 2. — eTable 1. 10-Year Overall Mortality and 10-Year Cancer-Specific Mortality in the Two Study Groups in Denmark and in Sweden eTable 2. Cox Proportional Hazards Regression for 10-Year Overall Mortality and 10-Year Colorectal Cancer–Specific Mortality in High-Frequency vs the Low-Frequency Groups eFigure. Patient Flow Through the COLOFOL Trial [file jamanetwopen-e2446243-s002.pdf]

Supplementary Online Content

Sørensen HT, Horváth-Puhó E, Petersen SH, Wille-Jørgensen P, Syk I; COLOFOL Study Group. More vs less frequent follow-up testing and 10-year mortality in patients with stage II or III colorectal cancer: secondary analysis of the COLOFOL randomized clinical trial. *JAMA Netw Open.* 2024;7(11):e2446243. doi:10.1001/jamanetworkopen.2024.46243

eTable 1. 10-Year Overall Mortality and 10-Year Cancer-Specific Mortality in the Two Study Groups in Denmark and in Sweden

eTable 2. Cox Proportional Hazards Regression for 10-Year Overall Mortality and 10-Year Colorectal Cancer–Specific Mortality in High-Frequency vs the Low-Frequency Groups

eFigure. Patient Flow Through the COLOFOL Trial

This supplementary material has been provided by the authors to give readers additional information about their work.

© 2024 Sørensen HT et al. *JAMA Network Open.*

eTable 1. 10-Year Overall Mortality and 10-Year Cancer-Specific Mortality in the Two Study Groups in Denmark and in Sweden

			**High-Frequency Group**	**Low Frequency Group**	
			**Risk (95% CI)**	**Risk (95% CI)**	**Risk difference (95% CI)**
**10-year overall mortality**	Intention-to-treat analysis	Denmark	29.25 (25.70-33.16)	29.36 (25.83-33.25)	0.11 (-5.16-5.38)
		Sweden	25.30 (22.15-28.81)	27.54 (24.27-31.15)	2.23 (−2.56-7.02)
	Per-protocol analysis	Denmark	28.86 (25.27-32.84)	29.04 (25.44-33.02)	0.18 (−5.18-5.54)
		Sweden	24.10 (20.90-27.69)	26.77 (23.43-30.47)	2.67 (−2.22-7.56)
**10-year colorectal cancer-specific mortality**	Intention-to-treat analysis	Denmark	16.81 (13.87-20.00)	16.23 (13.36-19.36)	-0.58 (−4.87-3.71)
		Sweden	14.48 (11.91-17.29)	15.69 (13.02-18.60)	1.21 (−2.67-5.09)
	Per-protocol analysis	Denmark	17.24 (14.22-20.51)	16.70 (13.72-19.93)	−0.54 (−4.97-3.89)
		Sweden	14.10 (11.47-16.99)	15.11 (12.39-18.07)	1.01 (−2.95-4.97)

© 2024 Sørensen HT et al. *JAMA Network Open.*

eTable 2. Cox Proportional Hazards Regression for 10-Year Overall Mortality and 10-Year Colorectal Cancer–Specific Mortality in High-Frequency vs the Low-Frequency Groups

		**Crude Hazard Ratio (95% CI)**	**Adjusted Hazard Ratio (95% CI)**
**10-year overall mortality**	Intention-to-treat analysis	0.94 (0.81-1.10)	0.93 (0.80-1.08)
	Per-protocol analysis	0.93 (0.80-1.09)	0.91 (0.78-1.07)
**10-year colorectal cancer-specific mortality**	Intention-to-treat analysis	0.96 (0.79-1.18)	0.96 (0.78-1.17)
	Per-protocol analysis	0.97 (0.79-1.19)	0.95 (0.77-1.17)

© 2024 Sørensen HT et al. *JAMA Network Open.*

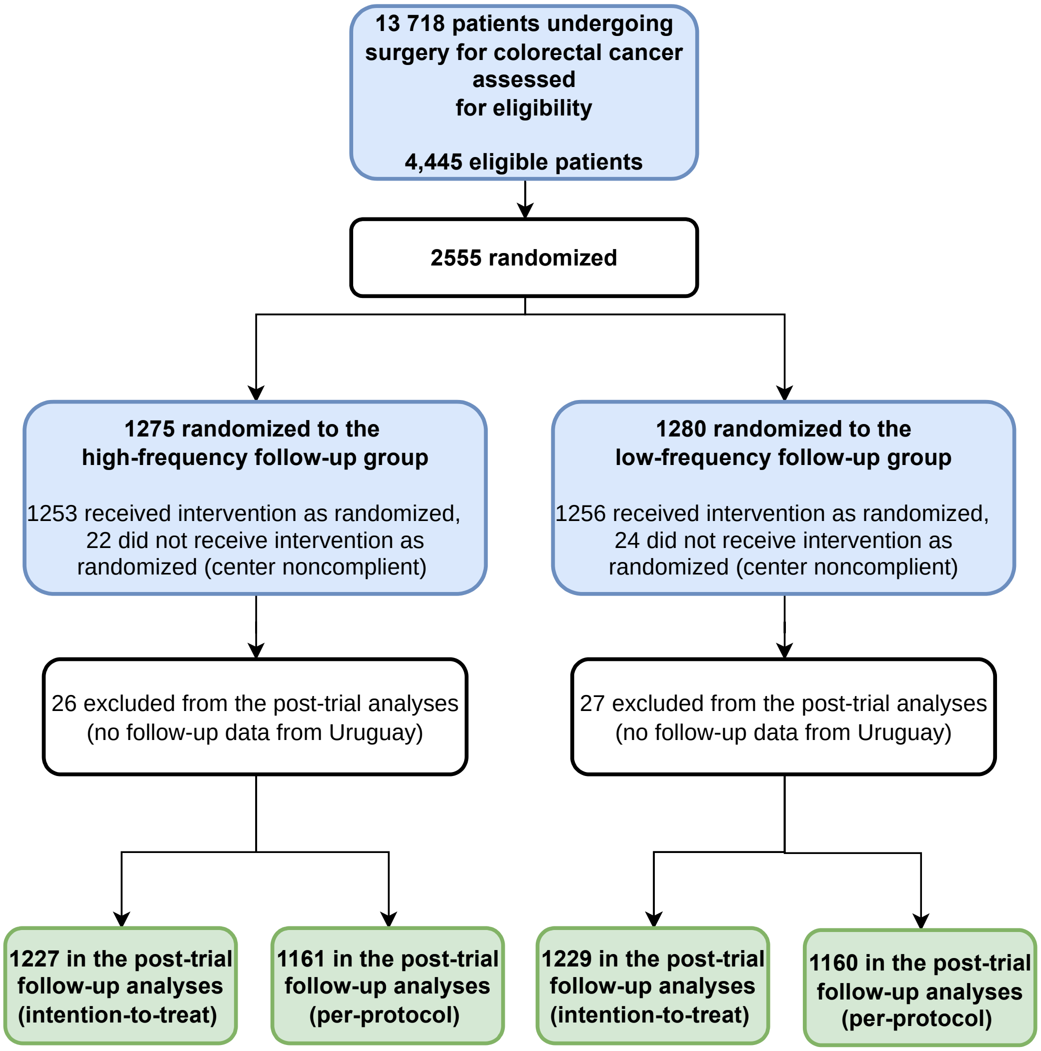

eFigure. Patient Flow Through the COLOFOL Trial

© 2024 Sørensen HT et al. *JAMA Network Open.*
