# Supplementary material for: More vs Less Frequent Follow-Up Testing and 10-Year Mortality in Patients With Stage II or III Colorectal Cancer: Secondary Analysis of the COLOFOL Randomized Clinical Trial
Source: JAMA Netw Open. 2024 Nov 21;7(11):e2446243. doi: 10.1001/jamanetworkopen.2024.46243 (PMC11582930; doi:10.1001/jamanetworkopen.2024.46243)
Supplement: Supplement 3. — Nonauthor Collaborators [file jamanetwopen-e2446243-s003.pdf]

Supplemental Online Content: Nonauthor Collaborators
*First name, last name, and suffix (if applicable) are required and will appear in PubMed.

***Group Name(s): COLOFOL Study Group**							
***First Name and Middle Initial(s)**	***Last Name**	***Suffix (eg, Jr, III)**	Academic Degrees	Institution	Location (city, state/province, country)	Role or Contribution, eg, chair, principal investigator	Group (if more than 1 Group listed in the byline) and/or Subgroup (eg, Steering Committee)
Søren	Laurberg		MD	Aarhus University Hospital	Denmark	Co-investigator for the initial study	COLOFOL STEERING COMMITTE for the initial study
Andrew	Renehan		MD	Univesity of Manchester	England	Co-investigator for the initial study	COLOFOL STEERING COMMITTE for the initial study
Kenneth	Smedh		MD	Västerås Hospital	Sweden	Co-investigator for the initial study	COLOFOL STEERING COMMITTE for the initial study
Per Vadgaard	Andersen		MD	Svendborg Hospital	Denmark	Co-investigator for the initial study	COLOFOL STUDY GROUP
Henrik	Christensen		MD	Aarhus University Hospital	Denmark	Co-investigator for the initial study	COLOFOL STUDY GROUP
Per	Gandrup		MD	Aalborg University Hospital	Denmark	Co-investigator for the initial study	COLOFOL STUDY GROUP
Per	Jess		MD	Hillerød Hospital	Denmark	Co-investigator for the initial study	COLOFOL STUDY GROUP
Mogens Rørbæk	Madsen		MD	Herning Hospital	Denmark	Co-investigator for the initial study	COLOFOL STUDY GROUP
Allan Gorm	Pedersen		MD	Randers Hospital	Denmark	Co-investigator for the initial study	COLOFOL STUDY GROUP
Erling	Østergaard		MD	Viborg Hospital	Denmark	Co-investigator for the initial study	COLOFOL STUDY GROUP
Pernilla Hansdotter	Andersson		MD	Skåne University Hospital, Malmö	Sweden	Co-investigator for the initial study	COLOFOL STUDY GROUP
Jonas	Bengtsson		MD	Sahlgrenska Hospital, Gothenburg	Sweden	Co-investigator for the initial study	COLOFOL STUDY GROUP
Mats	Bragmark		MD	Danderyd University Hospital	Sweden	Co-investigator for the initial study	COLOFOL STUDY GROUP
Pamela	Buchwald		MD	Helsingborg Hospital	Sweden	Co-investigator for the initial study	COLOFOL STUDY GROUP
Monica	Egenvall		MD	Karolinska Hospital Huddinge, Stockholm	Sweden	Co-investigator for the initial study	COLOFOL STUDY GROUP
Parastau	Farahnak		MD	Södersjukhuset, Stockholm	Sweden	Co-investigator for the initial study	COLOFOL STUDY GROUP
Joakim	Folkesson		MD	Uppsala Academic Hospital	Sweden	Co-investigator for the initial study	COLOFOL STUDY GROUP
Michael	Goldinger		MD	St. Görans Hospital, Stockholm	Sweden	Co-investigator for the initial study	COLOFOL STUDY GROUP
Rolf	Heuman		MD	Mora Hospital	Sweden	Co-investigator for the initial study	COLOFOL STUDY GROUP
Kenneth	Lindberg		MD	Södertälje Hospital	Sweden	Co-investigator for the initial study	COLOFOL STUDY GROUP
Anna	Martling		MD	Karolinska Hospital Solna, Stockholm	Sweden	Co-investigator for the initial study	COLOFOL STUDY GROUP
Pia	Näsvall		MD	Sunderby Hospital Luleå	Sweden	Co-investigator for the initial study	COLOFOL STUDY GROUP
Johan	Ottosson		MD	Kristianstad Hospital	Sweden	Co-investigator for the initial study	COLOFOL STUDY GROUP
Birger	Sandzén		MD	Norrland University Hospital, Umeå	Sweden	Co-investigator for the initial study	COLOFOL STUDY GROUP
Carlos	Barberousse		MD	Maciel University Hospital, Montevideo	Uruguay	Co-investigator for the initial study	COLOFOL STUDY GROUP
