# Supplementary material for: More vs Less Frequent Follow-Up Testing and 10-Year Mortality in Patients With Stage II or III Colorectal Cancer: Secondary Analysis of the COLOFOL Randomized Clinical Trial
Source: JAMA Netw Open. 2024 Nov 21;7(11):e2446243. doi: 10.1001/jamanetworkopen.2024.46243 (PMC11582930; doi:10.1001/jamanetworkopen.2024.46243)
Supplement: Supplement 4. — Data Sharing Statement [file jamanetwopen-e2446243-s004.pdf]

Data Sharing Statement

Sørensen. More vs Less Frequent Follow-Up Testing and 10-Year Mortality in Patients With Stage II or III Colorectal Cancer. *JAMA Netw Open*. Published November 21, 2024. doi:10.1001/jamanetworkopen.2024.46243

Data
Additional Information: ClinicalTrials.gov NCT00225641
Data available: No
